# Cortical and trabecular bone structure of the hominoid capitate

**DOI:** 10.1111/joa.13437

**Published:** 2021-05-04

**Authors:** Emma E. Bird, Tracy L. Kivell, Matthew M. Skinner

**Affiliations:** ^1^ Skeletal Biology Research Centre School of Anthropology and Conservation University of Kent Canterbury UK; ^2^ Department of Human Evolution Max Planck Institute for Evolutionary Anthropology Leipzig Germany

**Keywords:** cancellous bone, functional morphology, locomotion, primates, wrist

## Abstract

Morphological variation in the hominoid capitate has been linked to differences in habitual locomotor activity due to its importance in movement and load transfer at the midcarpal joint proximally and carpometacarpal joints distally. Although the shape of bones and their articulations are linked to joint mobility, the internal structure of bones has been shown experimentally to reflect, at least in part, the loading direction and magnitude experienced by the bone. To date, it is uncertain whether locomotor differences among hominoids are reflected in the bone microarchitecture of the capitate. Here, we apply a whole‐bone methodology to quantify the cortical and trabecular architecture (separately and combined) of the capitate across bipedal (modern *Homo sapiens*), knuckle‐walking (*Pan paniscus*, *Pan troglodytes*, *Gorilla* sp.), and suspensory (*Pongo* sp.) hominoids (*n* = 69). It is hypothesized that variation in bone microarchitecture will differentiate these locomotor groups, reflecting differences in habitual postures and presumed loading force and direction. Additionally, it is hypothesized that trabecular and cortical architecture in the proximal and distal regions, as a result of being part of mechanically divergent joints proximally and distally, will differ across these portions of the capitate. Results indicate that the capitate of knuckle‐walking and suspensory hominoids is differentiated from bipedal *Homo* primarily by significantly thicker distal cortical bone. Knuckle‐walking taxa are further differentiated from suspensory and bipedal taxa by more isotropic trabeculae in the proximal capitate. An allometric analysis indicates that size is not a significant determinate of bone variation across hominoids, although sexual dimorphism may influence some parameters within *Gorilla*. Results suggest that internal trabecular and cortical bone is subjected to different forces and functional adaptation responses across the capitate (and possibly other short bones). Additionally, while separating trabecular and cortical bone is normal protocol of current whole‐bone methodologies, this study shows that when applied to carpals, removing or studying the cortical bone separately potentially obfuscates functionally relevant signals in bone structure.

## Graphical abstract

This research examines the trabecular and cortical architecture of the capitate of great apes. It is concluded that the internal structure of the human capitate is differentiated from nonhuman apes by a combination of thin cortical bone in the distal segment and more anisotropic trabeculae in the proximal. Further, there are significant differences in the proximal and distal segments of the capitate which indicates variable loading and functional adaptation across the small bone in hominoids.

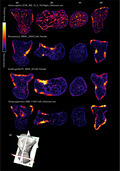

## 1 INTRODUCTION

Primates use their hands in a diverse set of postures to manipulate and navigate their environment (Fragaszy & Crast, 2016). The many articulations within the wrist are central to the capacity of the hand to move through multiple planes of space and, in combination with soft tissue morphology, joint congruence determines the degree of stability, flexibility and dexterity within the wrist and hand (Orr, 2010). The capitate articulates proximally with the scaphoid and lunate and distally with the trapezoid, hamate, and metacarpals 2, 3, and, sometimes, 4 (Kivell, 2016a). As such, the external morphology of the capitate plays a key role in the range of motion at the wrist as it is a central component of the midcarpal joint proximally and the carpometacarpal joints distally (Crisco et al., 2005; Jenkins & Fleagle, 1975; Jouffroy & Medina, 2002; Kijima & Viegas, 2009; Lewis, 1989; Orr, 2017; Orr et al., 2010).

The external morphology of the hominoid capitate has featured in hypotheses about the locomotor behavior in the last common ancestor of *Pan* and Hominini (Begun, 2004; Dainton & Macho, 1999; Kivell & Schmitt, 2009; Richmond et al., 2001; Tocheri et al., 2007) and the evolution of hominin dexterity and tool‐related behaviors (Marzke, 1983; Niewoehner et al., 1997; Rein & Harvati, 2013; Wolfe et al., 2006). However, drawing locomotor or postural inferences based on external morphology is potentially confounded by the retention of primitive features that are no longer functionally significant (Kivell, 2016b; Lieberman, 1997; Pontzer et al., 2006; Ruff et al., 2006; Ward, 2002; Zeininger et al., 2011). Furthermore, making biomechanical inferences from external morphology requires in‐depth knowledge of the form‐function relationship of the bone as well as its surrounding soft tissue and articular environment. This is a particular challenge for carpal research as understanding the kinematics and kinetics of the wrist is inherently difficult due to complications in imaging and analyzing the small, closely compacted bones without disrupting the normal kinematic chain (Brainerd et al., 2010; Gatesy et al., 2010; Orr, 2016; Wolfe et al., 2000, 2006). Although advances in 3D imaging and computational techniques have begun to improve our knowledge of human wrist biomechanics (see Orr, 2016 for review), our understanding of nonhuman ape biomechanics remains more limited (but see Orr, 2017, 2018). Moreover, the functional relationship between cortical and trabecular tissue within short bones is not well understood, and it is not clear how they may interact to provide whole bone functionality under the high mechanical loads of locomotion and manipulation. To date, it has yet to be determined whether the internal bone structure of the capitate might reflect differences in hand and wrist use across extant hominoids. Here we apply a whole‐bone methodology to investigate how the internal cortical and trabecular bone structure potentially varies within the capitate in a broad sample of *Homo* (recent humans), *Pan* (chimpanzees and bonobos), *Gorilla*, and *Pongo* (orangutans).

### 1.1 Trabecular bone: The relationship between behavior and structure

In addition to some important clade specific synapomorphies (Lewis, 1989; Sarmiento, 1988; Tocheri et al., 2008), the wrists of extant hominoids are adapted to their specialized behaviors and are habitually loaded in different ways. The *Homo* hand is conspicuous among the ape clade as the only species not to habitually utilize the hand for locomotion. Instead, the wrist experiences forces generated predominantly during tool use and other forms of manipulation. High compressive loads are imposed across the wrist by muscle contractions arising from the strong and forceful human thumb as well as flexion of the fingers (Bardo et al., 2018; Marzke, 1997; Napier, 1956; Tocheri, 2007). Bones must also withstand and transmit sheer and tensional strains as force is transferred radio‐ulnarly across the wrist (Marzke, 2013; Tocheri, 2007). There is an abundance of clinical evidence to support the hypothesis that the Dart Throwers Motion (DTM) is the functional axis of human wrist movement (Brigstocke et al., 2014; Crisco et al., 2005, 2011; Schuind et al., 1994). The DTM runs from radial deviation in extension to ulnar deviation in flexion and is used across numerous activities from throwing an object to pouring water from a jug (Brigstocke et al., 2014). During this movement, the capitate is very mobile against a stabilized proximal row, with the rotation axis perpendicular to the wrist movement (Crisco et al., 2005).

In contrast, nonhuman apes utilize their forelimbs during locomotion. *Pongo* utilize a range of torso orthograde suspensory and climbing postures in an almost exclusively arboreal environment (Manduell et al., 2011; Thorpe & Crompton, 2006, 2009). In these positions, the wrist experiences substantial tensile loading from gravitational forces and stabilising ligaments, as well as compressive stress from muscle contractions (Isler & Thorpe, 2004; Swartz et al., 1989). *Gorilla* and *Pan* are primarily terrestrial knuckle‐walkers but also engage in various types and frequencies of arboreal locomotion depending on the species, population or sex (Doran, 1993; Hunt, 1992; van Lawick‐Goodall, 1968; Neufuss et al., 2017; Remis, 1995, 1998; Thompson et al., 2018). During knuckle‐walking, the wrist must resist compressive loading from both muscle contractions stabilizing the joints and gravitation forces acting on the body mass (Carlson & Patel, 2006). However, the knuckle‐walking posture differs somewhat between the two genera. When compared to *Gorilla*, *Pan* typically use more variable hand and forelimb postures, do not bear weight as evenly across the digits, and more frequently engage a palm‐in forelimb posture (Finestone et al., 2018; Inouye, 1994; Matarazzo, 2013; Wunderlich & Jungers, 2009). *Gorilla* typically knuckle‐walk on digits 2–5 and more regularly utilize a palm‐backwards forelimb posture (Inouye, 1994; Matarazzo, 2013; Tuttle, 1969), although hand postures in the wild are more variable (Thompson et al., 2018). Although *Gorilla* are hypothesised to use a more neutral, columnar wrist posture than *Pan* (Kivell & Schmitt, 2009), recent kinematic studies of captive African apes found that *Gorilla* and *Pan* were generally similar in their degree of wrist of extension during knuckle‐walking (Finestone et al., 2018; Thompson, 2020).

Bone functional adaptation describes the biological process of bone altering its structure to optimize resistance against peak mechanical loads habitually experienced throughout the lifetime of the individual (Barak et al., 2011; Doube et al., 2011; Martin et al., 1998; Ruff et al., 2006). Numerous experimental studies suggest that variation in structure reflects, at least in part, load experienced during life (see Kivell, 2016b for review) and thus provides an opportunity to draw behavioral inferences better linked to actual, rather than potential, behavior (Frost, 1987; Ruff & Runestad, 1992). Bone functional adaptation research cannot only facilitate a greater understanding of the joint loading and kinematics of extant species but may also provide an informative avenue for behavioral reconstruction in fossil taxa (DeSilva & Devlin, 2012; Dunmore et al., 2020; Georgiou et al., 2020; Griffin et al., 2010; Kivell et al., 2018; Skinner et al., 2015; Su & Carlson, 2017). Previous studies of primate trabecular bone structure within the capitate have used a volume of interest (VOI) sampling sphere but have found limited functional correlation with locomotor behavior (Ragni, 2020; Schilling et al., 2014). However, using a whole epiphysis/bone methodology has been more functionally informative for hand bone studies (Dunmore et al., 2019; Stephens et al., 2016, 2018; Tsegai et al., 2013, 2017). Furthermore, a whole‐bone approach to carpal functional adaptation is preferable given their irregular shapes and variation across different taxa (Gross et al., 2014; Schilling et al., 2014; Tsegai et al., 2013).

However, inferring a form‐function relationship between bone microarchitecture and behavior is not always straightforward due to several potentially confounding variables (for a comprehensive review and discussion see Kivell, 2016b). Firstly, bone modelling (*sensu* Barak, 2019) is influenced by the genetic blueprint of the individual, as well as life history factors such as lactation or pregnancy (Kalkwarf & Specker, 1995; Lieberman, 1996; Lovejoy et al., 2003; Parsons et al., 1997; Paternoster et al., 2013; Pettersson et al., 2010; Tsegai et al., 2017; Yeni et al., 2011). Systemic features such as these potentially undermine our ability to differentiate between functional and nonfunctional patterns expressed in bone structure across different individuals or species. Secondly, there is a higher capacity for functional adaptation to occur during the juvenile and young adult periods and the extent to which bone microarchitectural patterns can be linked to adult behavior has been debated (Bertram & Schwartz, 1991; Pearson & Lieberman, 2004; Ruff et al., 2006). This is particularly salient when analyzing African apes because locomotor behavior is known to differ across age categories (Doran, 1992, 1997). Finally, there is also uncertainty regarding the loading frequency and magnitude necessary to induce a functional adaptation response (Barak et al., 2011; Frost, 1987; Ruff et al., 2006; Wallace et al., 2015). Consequently, microarchitecture will never represent the mechanical ideal of the bone as competing demands on bone tissue will result in a compromise morphology (Ruff et al., 2006).

### 1.2 Cortical bone: Contribution to bone structure and functional adaptation

Carpal bones function within an intricate biomechanical environment. The bones and ligaments are interdependent and work together making minor adjustments and movements in concert to create overall hand motion (Kijima & Viegas, 2009; Lewis, 1989; Orr, 2017). Among the carpus, the central role of the capitate within the midcarpal joint and its articulation with the metacarpus makes it an ideal bone to investigate functional differences in wrist loading. The distal capitate is not only compressed via its carpometacarpal articulations but it also receives tensional strain via the attachment of several extrinsic (between carpals and other hand bones) and intrinsic (between carpal bones) ligaments (Kijima & Viegas, 2009; Regal et al., 2020; Schuind et al., 1995). In contrast, the proximal capitate does not receive any ligaments but forms the “ball” component of the ball and socket midcarpal joint within the highly mobile proximal row and is thus loaded predominantly in compression (Garcia‐Elias et al., 1994; Kivell, 2016a; Lewis, 1989; Orr, 2017).

Unlike long bones, short bones like carpals generally have a thin cortical shell and the entire internal space is filled with trabeculae (Currey, 2002; Schilling et al., 2014). During movement, short bones are likely to bear a significant portion of the load imposed upon the region as they resist against compressive forces and transfer load through the bone from one joint articulation to another, while also being strained via tensional loads from attached ligaments (Currey, 2002; Yao et al., 2020). Cortical and trabecular bone have divergent material properties due to differences in porosity, mineralization and cellular constitution (Currey, 2002). Cortical bone is stiffer and stronger than trabecular bone (Guo, 2001; Martin et al., 1998), but due to its lower porosity, it is slower than trabecular bone to model and is less compliant (Hart et al., 2017; Martin et al., 1998). While the two tissues work together to create the functionality of the whole bone, their relative contributions to stiffness, strength and homeostasis differs across regions of the same bone (Barak et al., 2010; Doube et al., 2009). It is not currently understood how cortical and trabecular bone work together to meet the mechanical demands of the carpus, particularly under the high mechanical demands of locomotion.

By quantifying the internal bone architecture of the hominoid capitate using a whole‐bone methodology, this study aims to investigate whether differences in trabecular and cortical architecture among hominoids may relate to the divergent hand use across the clade. We also examine the proximal and distal segments of the capitate separately, due to the differences in the soft tissue and articular relationships with the surrounding bones.

### 1.3 Allometry: Body size and bone structure

As functional adaptation research aims to identify markers of behavior rather than body size, analyzing bone parameters for allometric effects has been integral to interspecific analyses (Ruff, 1984). Decades of research across various species has yet to find consistent patterns; however, some research suggests there may be a general pattern across mammals and birds whereby bone volume to total volume (BV/TV) and degree of anisotropy (DA) are independent of body mass (Barak et al., 2013; Christen et al., 2015; Cotter et al., 2009; Doube et al., 2011; Kivell et al., 2018; Komza & Skinner, 2019; Schilling et al., 2014; Tsegai et al., 2017) while trabecular thickness (Tb.Th), trabecular number (Tb.N) and trabecular separation (Tb.Sp) scale with negative allometry (Barak et al., 2013; Kivell et al., 2018; Ragni, 2020; Ryan & Shaw, 2013). Cortical thickness (Ct.Th) is often reported to be isometric or slightly positively allometric (Demes et al., 2000; Fajardo et al., 2013; Runestad, 1997). However, not all studies find BV/TV and DA to be independent of body mass (for example Fajardo et al., 2013; Mielke et al., 2018; Ragni, 2020; Ryan & Shaw, 2013) nor the negative relationship with Tb.Th, Tb.N and Tb.Sp (for example Doube et al., 2011; Fajardo et al., 2013; Komza & Skinner, 2019; Tsegai et al., 2017) Few allometric studies have been undertaken on short bones. Tsegai et al. (2017) found no correlation between trabecular parameters or Ct.Th with size in intraspecific analyses of the *Homo* and *Pan* talus. Similarly, an interspecific analysis in Schilling et al. (2014) of the primate capitate found only Tb.N to scale with negative allometry. Ragni (2020) found a greater number of significant relationships within the capitate of *Pan* and *Gorilla* with Tb.Th, Tb.N, and Tb.Sp showing negative allometry and DA and BV/TV expressing isometry. These conflicting results may be due in part to methodological differences for sampling trabeculae or calculating size. Nevertheless, the effects of allometry on the hominoid capitate remain unclear.

### 1.4 Hominoid capitate morphology

#### 1.4.1 Distal capitate

In all hominoids, the distal capitate is bound to the surrounding bones via strong ligaments which are often described as a unit that moves in unison during extension and flexion (Crisco et al., 2005; Moojen et al., 2003; Orr, 2010; Richmond, 2006; Richmond et al., 2001; Tang et al., 2011). The capitate articulates distoradially with the trapezoid (although this articulation can be absent in *Gorilla*) and second metacarpal (Mc2), and distally with the third and sometimes fourth metacarpals (Kivell, 2016a; Lewis, 1989). The topology of the metacarpal joint surfaces in the distal row is more complex and irregular in *Pan* and *Gorilla* compared to the smoother surfaces in *Pongo*; however, the capacity for extension is linked to the range of movement at the midcarpal joint rather than at the carpometacarpal junction (Begun, 2004; Orr, 2017; Richmond et al., 2001). The distal capitate in modern *Homo sapiens* is considered to have several derived features linked to committed manipulation and increased efficiency of radio‐ulnar force transfer (Tocheri, 2007; Tocheri et al., 2008). A distally oriented capitate‐Mc2 articulation allows pronation of the second finger towards the thumb facilitating precision grip, while a palmarly positioned and expanded capitate‐trapezoid articulation is thought to better resist high radio‐ulnarly oriented forces incurred by the thumb during tool‐related activities (Marzke, 1997; Tocheri, 2007; Tocheri et al., 2008). Furthermore, the disto‐dorso‐radial corner is truncated to accommodate the third metacarpal (Mc3) styloid process, providing a stable joint for transmitting high forces and resisting subluxation of the third ray during tool use (Marzke & Marzke, 1987; Niewoehner et al., 1997; Riley & Trinkaus, 1989; Tocheri et al., 2008; Ward et al., 2014). In nonhuman apes, load transfer also occurs radio‐ulnarly as bones of the distal carpal row are compressed against one another. However, in contrast to humans, the orientations of the articular surfaces of the capitate (and distal carpal row more generally) indicate the wrist is better adapted to withstand and transfer proximo‐distally oriented forces, which arise during use of the forelimb in locomotion (Tocheri, 2007; Tocheri et al., 2008). Only a small proportion of the dorsal surface of the distal capitate is without articular surfaces. In this distal segment, compression is induced at the distal, radial and ulna articular surfaces, while tension is induced by the supporting intrinsic ligaments surrounding these articulations. Tension further arises from the several intrinsic and extrinsic ligaments attached to the palmar and dorsal surfaces (Kijima & Viegas, 2009; Regal et al., 2020).

#### 1.4.2 Proximal capitate

**FIGURE 1:**
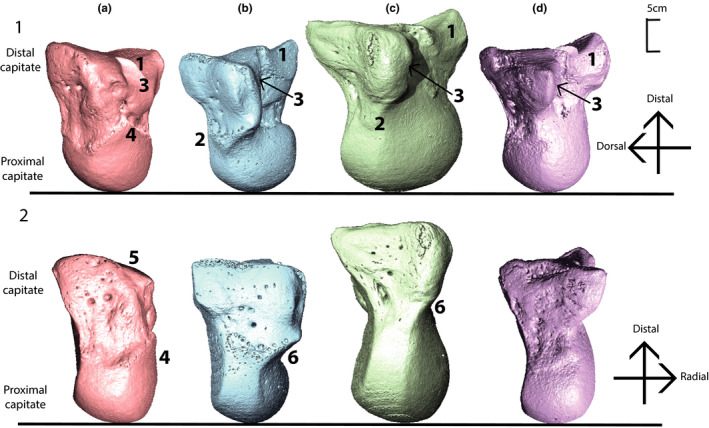
CT‐derived surface models of a left capitate from each genus showing variation in external morphology. Capitates have been scaled to relative size. Rows: (1) Capitates are oriented dorso‐radially and (2) capitates are oriented dorsally. Columns: (a) *Homo sapiens* (DCW_AM_10_0_182), (b) *Pan troglodytes* (SMF_4104), (c) *Gorilla gorilla* (ZMB_83587), (d) *Pongo pygmaeus* (ZMB_6948). Numbers representing anatomical features: “1” Mc2 articulation, “2” Dorsal ridge, “3” trapezoid articulation, “4” radial‐palmar expansion of the proximal capitate, “5” Mc3 styloid process articulation, “6” waisted mid‐capitate

In great apes, the rounded proximal surface of the capitate articulates with the bones of the proximal row to form the crux of the midcarpal joint (Kivell, 2016b). No ligaments attach directly onto the proximal capitate thus compared to the distal row, the bones of the midcarpal joint are able to move more independently of one another (Crisco et al., 2005; Kijima & Viegas, 2009; Moojen et al., 2003; Regal et al., 2020). In *Pongo*, the proximal capitate is radio‐ulnarly narrow in comparison to the other great apes (Figure 1; Richmond et al., 2001). Notably, the os centrale is not fused to the scaphoid as it is in the other hominids and thus excludes the scaphoid from articulating with the capitate resulting in relatively greater freedom of movement at the midcarpal joint (Begun, 2004; Orr, 2018). In *Pan* and *Gorilla*, the proximal capitate is enlarged on the radial aspect, which produces a “waisted” mid‐region forming an embrasure with the trapezoid (Kivell, 2016a; Orr, 2018; Richmond et al., 2001; Wolfe et al., 2006). There is also a notable radio‐ulnar ridge along the distal extent of the dorsal articular surface that extends onto the hamate (Richmond et al., 2001). These features contribute to the so called “screw‐clamp mechanism” that describes the functional complex limiting extension at the midcarpal joint. During extension, the scaphoid is wedged in between the capitate and trapezoid, providing stability between the proximal and distal row (Jenkins & Fleagle, 1975; Orr, 2005, 2017; Richmond, 2006; Richmond et al., 2001; Tuttle, 1969). *Homo* also exhibits the fused scaphoid‐os centrale and radially expanded proximal capitate; however, an enlargement of the bone in the radial‐palmar region results in a less dramatic “waisting” of the bone, resulting in a range of extension intermediate between the other hominoids (Lewis, 1977; Lewis, 1989; Orr, 2017). Notably, the proximal capitate is the crux of the functional axis of the DTM (Crisco et al., 2005). During motion, the rotation axis of the capitate is perpendicular to the movement of the DTM as it moves across a virtually motionless scaphoid and lunate (Crisco et al., 2005). Thus, although a small bone, the proximal and distal portion of the capitate functions within notably different ligamentous and articular environments.

### 1.5 Hypotheses

This research centers on three interrelated hypotheses for both trabecular and cortical bone that are summarized in Table 1 and elaborated below.

**TABLE 1:** Summary of the hypotheses, predictions, and statistical tests used in this study

Hypothesis	Predictions	Statistical tests
#1		
Locomotor and behavioral differences among extant hominoids will result in significantly different trabecular and cortical architecture in their capitates	Between species	
	Knuckle‐walking taxa will exhibit high BV/TV and DA *Pongo* will show intermediate BV/TV and low DA *Homo* will exhibit low BV/TV and intermediate DA Cortical bone will be thickest in *Gorilla* and *Pan*, followed by *Pongo*, then *Homo*.	Kruskal‐Wallis one‐way ANOVA Pairwise Wilcoxon rank‐sum tests
#2		
Proximal and distal segments will show significantly differentiated internal bone architecture	Between Species	
	Distal to proximal ratios will be statistically undifferentiated among the study taxa	Wilcoxon signed‐rank test
	Within Species	
	The distal aspect will have higher BV/TV and DA compared to the proximal aspect across all species The distal cortex will be significantly thicker than the proximal across all species	Kruskal‐Wallis one‐way ANOVA Pairwise Wilcoxon rank‐sum test
#3		
Allometry	Between species	
	Only Tb.N will show a significant negative relationship to body size, while all other parameters will be uncorrelated	Reduced major axis regression
	Within species	
	No parameters will exhibit significant correlations with body size	Reduced major axis regression

### 1.6 Trabecular bone architecture

We predict that the capitate of knuckle‐walking *Gorilla* and *Pan* will have high relative BV/TV and high DA (Table 1, Hypothesis 1) due to the presumed high compressive forces and reduced mobility from their more extension‐limiting midcarpal joint. In contrast, we predict that the *Pongo* capitate will have intermediate BV/TV and low DA due to their predominantly suspensory behavior, resulting in reduced compression but greater mobility. We expect *Homo* to exhibit low BV/TV and intermediate DA because their capitate is not loaded during locomotion and presumably has the least compressive loading but more predictable movement along the DTM axis.

Given the differences in mobility and presumed loading between the proximal and distal portions of the capitate, we hypothesize that there will be differences in the trabecular bone structure between these segments (measured as ratios). It is predicted that the distal aspect will have higher BV/TV and DA compared to the proximal aspect across all species (Table 1, Hypothesis 2). As there are no previous studies that have addressed this question for the capitate, we test the null hypothesis that these ratios will be similar among the study taxa. Although we report Tb.Th, Tb.N and Tb.Sp, we do not make explicit predictions about these parameters because all contribute, potentially in a variety of different combinations, to BV/TV.

### 1.7 Cortical bone thickness

The contribution of cortical bone to the functional adaptation of the capitate in hominoids has never been investigated. Given the assumed loading differences described above, we predict that the cortical bone will be thickest in *Gorilla* and *Pan*, followed by *Pongo*, with *Homo* exhibiting the thinnest cortex (Table 1, Hypothesis 1). Also following the predictions for trabecular bone, it is predicted that the cortex of the distal capitate should be significantly thicker than the proximal capitate for all genera.

In long bones, the joint surface tends to have a thin layer of cortical bone covering a dense trabecular network that transfers load towards the thicker and stronger diaphyseal cortex (Currey, 2002). In short bones, the cortex is similarly described as thin; however, the relationship between cortical and trabecular bone has never been quantified among hominoids. Additionally, it is unclear whether the behavioral differences among ape genera will result in different ratios of cortical to trabecular bone. Therefore, this study will investigate the relative contribution of cortical bone to total bone volume, testing the null hypothesis that these ratios will be similar among the study taxa (Table 1, Hypothesis 2).

### 1.8 Interspecific and intraspecific allometry in internal bone structure

As this study incorporates hominoids of diverse body size, interspecific and intraspecific allometry is also investigated. Predictions are outlined in Table 1 (Hypothesis 3) and follow the results of Schilling et al. (2014) for the interspecific and Tsegai et al. (2017) for the intraspecific predictions.

## 2 MATERIALS AND METHODS

### 2.1 Sample

The study sample includes capitates (*n* = 69) from *Homo sapiens* (*n* = 26), *Pan troglodytes* and *Pan paniscus* (*n* = 14), *Gorilla* sp. (*n* = 16) and *Pongo* sp. (*n* = 13) (Table 2 and Table S1). These taxa are categorized into three behavioral groups based on their most frequent locomotor behaviors: bipedal (*Homo*) knuckle‐walking (*Gorilla* and *Pan*) and suspensory (*Pongo*). Capitates from nonhuman apes were wild‐shot adults with no obvious signs of pathology. Consideration was given to ensuring a sex balance for each taxon when possible; however, 16 specimens had unknown sex.

**TABLE 2:** Summary of study sample

Taxon	*N*	Side		Sex			Behavioral group
		Right	Left	Female	Male	Unknown	
*Homo sapiens*	26	14	12	5	9	12	Bipedal/manipulative
*Pan paniscus*	8	5	3	4	4		Knuckle‐walking
*Pan troglodytes*	6	3	3	3	3		Knuckle‐walking
*Gorilla beringei*	1		1			1	Knuckle‐walking
*Gorilla gorilla*	15	8	7	7	7	1	Knuckle‐walking
*Pongo abelii*	2	1	1	1	1		Suspensory
*Pongo pygmaeus*	11	6	5	5	4	2	Suspensory

### 2.2 Computed tomography

Capitate specimens were scanned with either a BIR ACTIS 225/300 high‐resolution microCT scanner or a Diondo D3 high‐resolution microCT scanner at the Department of Human Evolution, Max Planck Institute for Evolutionary Anthropology, Germany, or a Nikon 225/XTH scanner at the Cambridge Biotomography Centre, University of Cambridge, United Kingdom. Specimens were scanned with an acceleration voltage of 100–160 kV and 100–140 μA using a 0.2‐ to 0.5‐mm copper or brass filter. Images were reconstructed as 16‐bit TIFF stacks. To ensure accurate post‐scan segmentation of thin trabeculae, scan resolution was limited to a maximum of 0.048 mm (average 0.032 mm) for nonhuman apes, and 0.035 mm (average 0.029 mm) for the *Homo* sample (Table S1). This resolution is below the suggested range for minimal error detection (Christen et al., 2016; Isaksson et al., 2011). Post‐scanning, each capitate was positioned into approximately the same orientation using Avizo 6.0 (Visualization Sciences Group, SAS). Segmentation of trabecular bone, including identification and removal of extraneous nonbone material, used the medical image analysis (MIA) clustering method (Dunmore et al., 2018). The MIA‐clustering method increases the reproducibility of results by reducing subjective input parameters required for other segmentation methods (Dunmore et al., 2018).

### 2.3 Data collection

**FIGURE 2:**
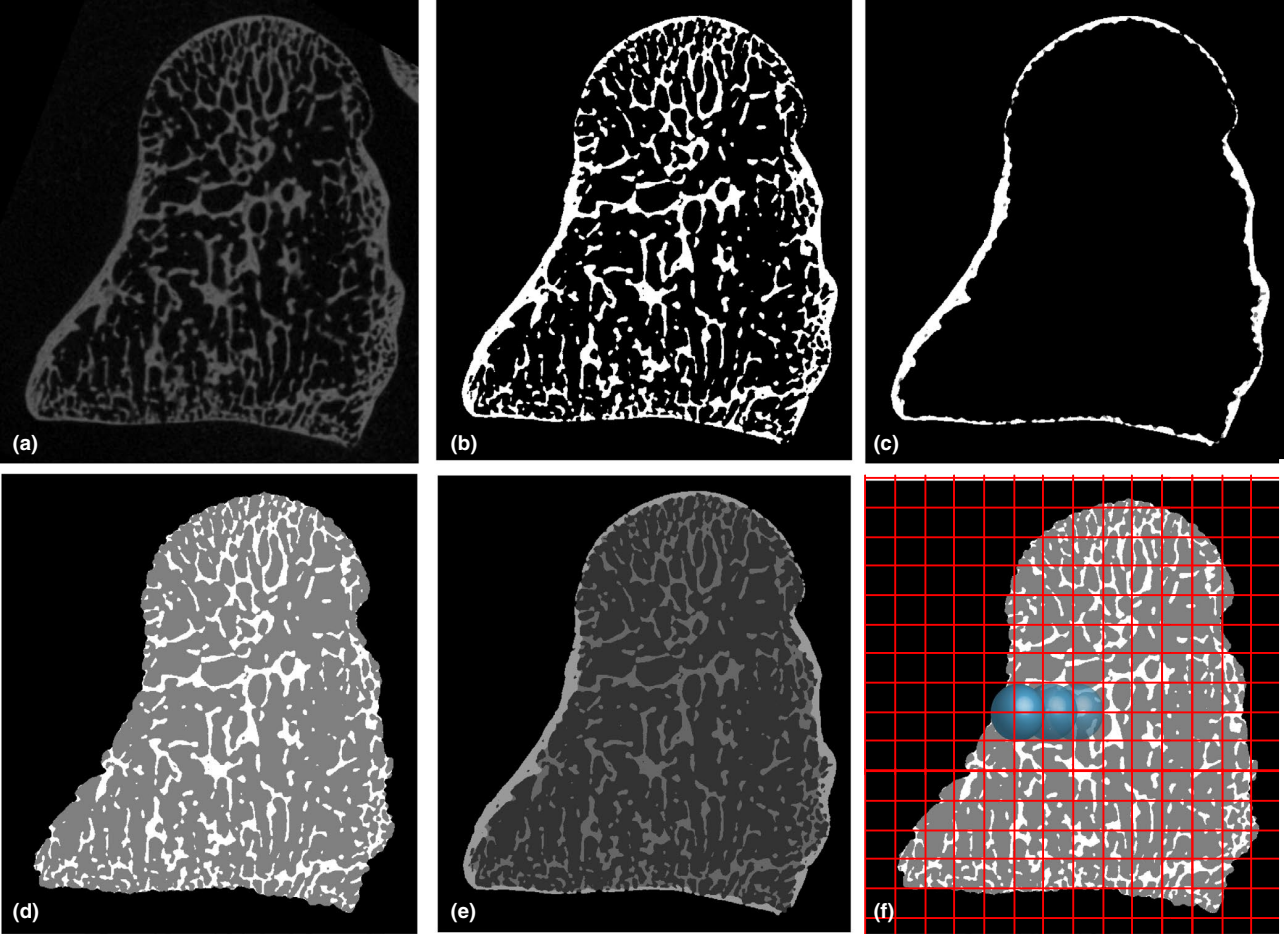
Images showing the morphological filters applied in medtool 4.3 for the whole‐bone analysis. (a) Original microCT of a *Homo sapiens* capitate, (b) microCT scan after MIA‐clustering segmentation, (c) cortical thickness image stack, allowing analysis of the cortex only, (d) trabecular bone image stack, allowing analysis of the trabeculae (white) only, (e) combined mask overlay, identifying cortical (lightest grey), trabecular (mid‐grey) and air (darkest grey internally and black externally) voxels, (f) sampling sphere (blue) moving across each node of the overlaid 3D grid (red) measuring bone parameters in the trabecular bone image stack

This study uses the medtool 4.3 software package (http://www.dr‐pahr.at/medtool/) to quantify bone parameters throughout the entire capitate utilizing the method outlined in Gross et al. (2014). In brief, medtool utilizes a series of morphological filters to identify the cortical, trabecular, internal (marrow), and background elements of the segmented CT scans. After MIA segmentation, medtool projects a series of rays onto outside of the bone (Figure 2b) that continue to move internally through the bone until a nonbone voxel is reached (Pahr & Zysset, 2009a). By using a value of average trabeculae thickness, morphological filters fill and close small holes present in the porous cortex allowing a smooth boundary contour between cortical and trabecular bone to be identified (Gross et al., 2014; Pahr & Zysset, 2009a, 2009b). Two *Gorilla*, one *Pan* and two *Pongo* specimens were excluded from the sample as the internal cortical‐trabecular boundary could not be confidently defined due to extreme cortical porosity (an example is provided in Figure S1). Medtool then superimposes the trabecular‐cortical boundary to the original image such that the pores within the cortex are maintained for analysis. Porosity is important to maintain within the cortical bone when quantifying microarchitecture as it has been linked to strength and elastic modulus (see Cooper et al., 2016 for review). Unique scalars are applied to the background, cortical, trabecular, and internal elements of the scan. A series of image stacks are created and include a cortex only stack (Figure 2c), trabecular and internal only stack (Figure 2d) and a trabecular and cortical combined stack (Figure 2e). A 3D grid with 2.5‐mm spaced nodes is then superimposed on an image stack and a 5‐mm sampling sphere moves from node to node to measure parameters across the entire bone (Figure 2f) (Pahr & Zysset, 2009a).

BV/TV is calculated as the ratio of bone to non‐bone voxels. DA is calculated via the mean intercept length (MIL) method (Whitehouse, 1974) and is calculated as 1—(min. eigenvalue/max. eigenvalue) which produces a number limited between 1 and 0, with 1 being complete anisotropy and 0 being complete isotropy. Tb.Th, Ct.Th, and Tb.Sp are computed in a similar way to the more well‐known BoneJ^®^ plugin (Doube et al., 2010) for ImageJ. Spheres are grown within the trabecular or cortical bone and medtool calculates the diameter of the largest sphere that fits within the bone (Hildebrand & Rüegsegger, 1997). For the calculation of Tb.Sp, medtool inverts the greyscale values of the image stack (Figure 2E) such that the “internal” voxels are now represented by the “bone” scalar. Similar to Tb.Th and Ct.Th, spheres are then grown within the internal voxels until a trabecular or cortical voxel is reached. The results of Tb.Sp and Tb.Th are used to calculate Tb.N using the formula 1/(Tb.Th + Tb.Sp).

**FIGURE 3:**
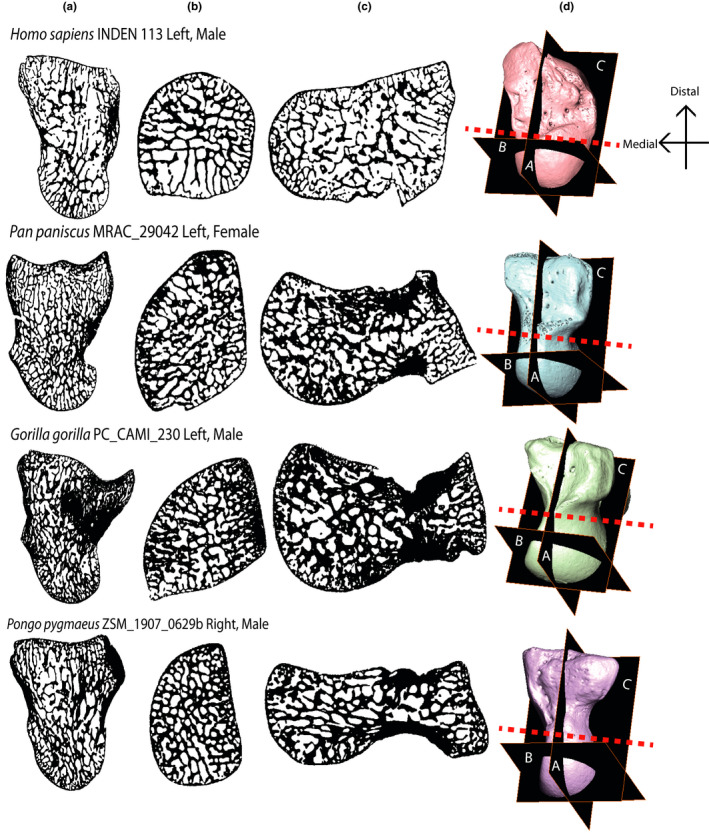
Three cross‐sections from the four study genera showing internal bone patterning. (a) *Y*–*Z* dimension, radial‐ulnar cross‐section, slice taken from mid‐section of bone. Distal is up; dorsal is left. (b) *X*–*Y* dimension, proximal‐distal cross‐section, slice taken from proximal mid‐capitate. Dorsal is up; radial is left. (c) *X*–*Z* dimension, dorsal‐palmar cross‐section, slice taken from midsection of bone. Ulnar is up; proximal is left. (d) Surface models of each bone showing the location of cross‐section (a, b, and c). The red dotted line indicates where capitates were partitioned into a distal and proximal VOI. Capitates are not to scale. Left capitates have been mirrored

Cortical and trabecular parameters were quantified in the whole capitate, as well as proximal and distal VOIs. To produce these VOIs, each capitate was cut just distal to the ulnar‐most point of the ridge delineating the extent of the lunate articulation on the dorsal proximal capitate, as per the measurement made in Richmond (2006) (Figure 3d). These VOIs are subjected to the same data collection process as outlined in Figure 2, quantifying all of the trabecular or trabecular and cortical bone within the proximal or distal segment. This delineation separates the proximal VOI as the section of the bone that does not contain any ligament attachment sites, from the distal VOI which does receive ligamentous attachments. To assess and compare the relative contribution of cortical bone to total bone volume, BV/TV was measured twice: firstly, in only the trabecular region of the bone (Figure 3d) as determined using medtool (see above) and, secondly, in the original MIA segmented specimen in which there is no partitioning between cortical and trabecular bone (Figure 2b). This provides a measure of BV/TV that combines cortical and trabecular bone (referred to as “total BV/TV” throughout). Relative thickness maps of Ct.Th and Tb.Th are generated by loading the Tb.Th output into ImageJ (1.50b) (https://imagej.nih.gov/ij/) and visualized using the 3D Volume Viewer plugin (http://rsb.info.nih.gov/ij/plugins/volume‐viewer.html ).

### 2.4 Statistical analysis

#### 2.4.1 Trabecular bone hypotheses

Mean differences in the proximal and distal trabecular parameters (trabecular BV/TV, DA, Tb.Th, Tb.N, Tb.Sp) were compared interspecifically using a Kruskal–Wallis one‐way ANOVA and pairwise Wilcoxon rank‐sum tests using the Holm p adjust method (R Core Team, stats package v3.6.1) (Table 1). A distal to proximal ratio was calculated for each parameter and a Wilcoxon signed‐rank test was applied within‐genus to test whether the mean values of the ratio were statistically significant. A Kruskal–Wallis one‐way ANOVA and pairwise Wilcoxon rank‐sum test examined interspecific differences in the ratios.

#### 2.4.2 Cortical bone hypotheses

To test for differences in cortical bone, mean differences in total BV/TV and Ct.Th were compared interspecifically in the proximal and distal segments using a Kruskal‐Wallis one‐way ANOVA and pairwise Wilcoxon rank‐sum tests using the Holm p adjust method (R Core Team, stats package v3.6.1).

Within each genus, a distal to proximal ratio was calculated for each parameter and a Wilcoxon signed‐rank test was applied to test whether mean values of the ratio were statistically significant. Additionally, we examined taxonomic differences in these ratios using a Kruskal‐Wallis one‐way ANOVA and pairwise Wilcoxon rank‐sum tests.

Two additional ratios were calculated in order to test for taxonomic differences in the relative proportion of cortical and trabecular bone. These ratios were compared between species, using a Kruskal‐Wallis one‐way ANOVA and pairwise Wilcoxon rank‐sum tests using the Holm p adjust method (R Core Team, stats package v3.6.1).

#### 2.4.3 Interspecific and intraspecific allometry

To test for allometric trends in the capitate, each whole‐bone cortical and trabecular parameter was interspecifically and intraspecifically analyzed in a reduced major axis regression (RMA). As a proxy for body mass, the volume (mm^3^) of each capitate was calculated in Paraview (4.8.2) using the Integrate Variables filter. The logged cube root of the volume was regressed against the logged bone parameters using the lmodel2 package in *R* (v1.7‐3). Interpretation follows Ryan and Shaw (2013); the shape parameters of BV/TV, DA and Tb.N will have an isometric slope equal to 0; values greater than 0 indicate positive allometry while values less than 0 are indicative of negative allometry. Size parameters, such as Ct.Th, Tb.Th and Tb.Sp will have an isometric slope of 1; positive allometry is indicated by a value greater than one and negative allometry by values of less than 1. All statistical tests conducted for hypotheses 1, 2 and 3 are considered significant if *p* ≤ 0.05.

## 3 RESULTS

### 3.1 Trabecular bone

Cross‐sections of each genera in Figure 3 provide an example of the internal structure of the capitate within three planes of view. The red dotted line in Figure 3d indicates where the capitate was partitioned into the proximal and distal VOIs.

### 3.2 Bone volume to total volume

**FIGURE 4:**
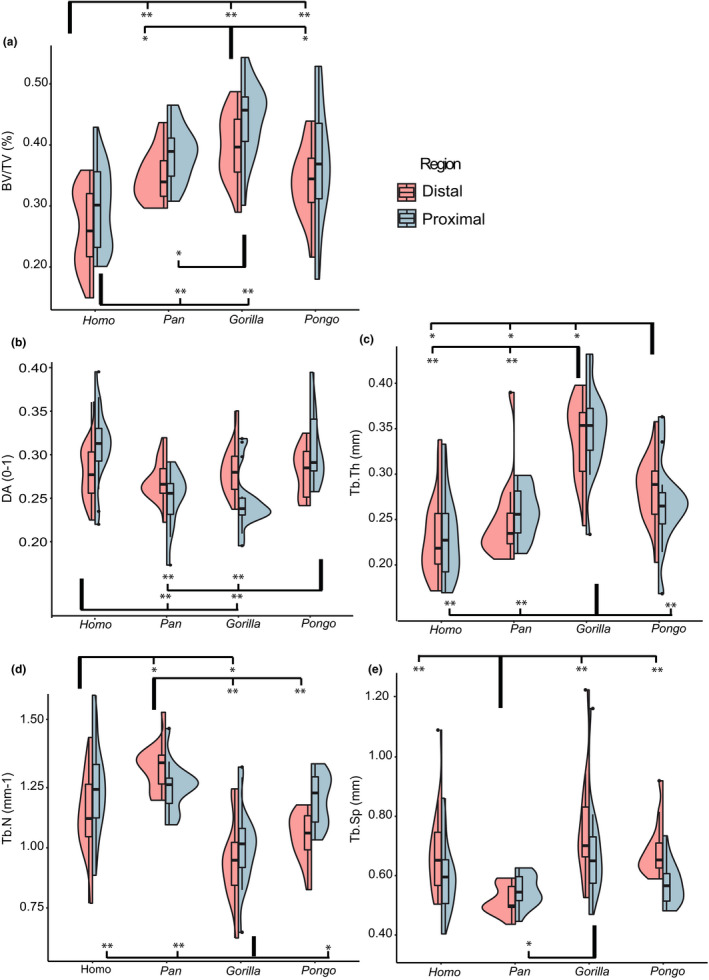
Split violin plots showing the distribution of trabecular results in the proximal and distal VOI of each genus. Images are generated using *ggplot2* in *R* (v. 1.2.1335) and utilize the default (Gaussian) kernel density estimation. Colored contours indicate the density of results across the data range. (a) Trabecular bone volume to total volume; (b) degree of anisotropy; (c) trabecular thickness; (d) trabecular number; (e) trabecular separation. Outliers are identified with ● and represent values 1.5 times above the fourth or below the first interquartile range. For all plots: significant pairwise comparisons are indicated by the square brackets for the distal VOI tests (top of graph) and proximal VOI tests (bottom of graph), *p ≤ 0.05; **p ≤ 0.001

Proximal and distal trabecular BV/TV differ significantly across the study sample (*p* ≤ 0.001 for both tests, Table S3). *Gorilla* has the highest proximal and distal BV/TV followed by *Pan*, then *Pongo*, with *Homo* having the lowest BV/TV values (Table S2). Proximally, pairwise comparisons show that *Pongo* is not differentiated from any other taxa, while other pairwise comparisons are significant. Distally, all pairwise comparisons are significant except between *Pongo* and *Pan* (Figure 4a, Table S3).

**FIGURE 5:**
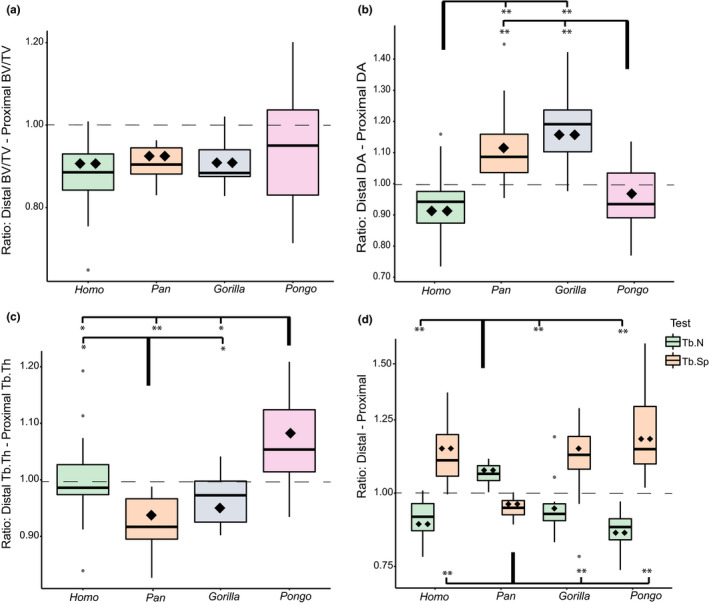
Boxplots of the five trabecular ratios for each genus as well as results for the intraspecific Wilcoxon signed‐rank test and interspecific pairwise rank‐sum tests. (a) Ratio of distal to proximal trabecular BV/TV; (b) ratio of distal to proximal DA; (c) ratio of distal to proximal Tb.Th; (d) ratio of distal to proximal Tb.N (green) and Tb.Sp (orange). For all figures: Values above the dotted line (ratio =1) indicate greater trabecular variable in the distal capitate. Significant pairwise comparisons of the ratios are indicated by the square brackets. For (d), the top brackets indicate the tests for Tb.N and the bottom brackets indicate those for Tb.Sp. *p ≤ 0.05; **p ≤ 0.001. Significant intraspecific Wilcoxon signed‐rank tests between the proximal and distal means are represented by the ♦ symbol thus indicate whether the difference between the mean distal and proximal trabecular variable was significantly different. ♦*p* ≤ 0.05; ♦♦*p* ≤ 0.001

Intraspecific comparisons of the BV/TV ratio (distal BV/TV relative to proximal BV/TV) reveal that all genera have greater trabecular BV/TV in the proximal aspect (Figure 5a; Tables S4 and S5). The differences between the two VOIs reach statistical significance in *Homo*, *Pan*, and *Gorilla* (*p* ≤ 0.001 for three tests; Table S4) but are nonsignificant in *Pongo*. The Kruskal‐Wallis test on the BV/TV ratio reveal that it does not differ significantly among the study sample (*p* = 0.429) indicating that although BV/TV differs between the proximal and distal capitate, the way it differs is similar among the hominoids.

### 3.3 Degree of anisotropy

Proximal DA differs significantly among the study sample (*p* ≤ 0.001); however, distal DA does not (*p* = 0.593, Table S3). Notably, DA is the only trabecular parameter which has a different significance result for the proximal and distal VOI. *Homo* and *Pongo* have the highest proximal DA with 0.30 followed by *Pan* and *Gorilla*, both with 0.24 (Figure 4b, Table S2). Distal DA differs by only 0.02 between the genera, with the highest value from *Gorilla* at 0.28 and lowest from *Pan* at 0.26 (Table S2). Pairwise comparisons reveal that proximally, *Homo* and *Pongo* are differentiated from *Pan* and *Gorilla* (*p* ≤ 0.001 for all four significant tests). Distally, there are no significant pairwise results (Figure 4b, Table S3).

Both *Gorilla* and *Pan* have a higher DA in the distal VOI whereas *Homo* and *Pongo* both have higher DA in the proximal and the difference between the proximal and distal VOIs is significant for all genera (Figure 5b, Table S4). The DA ratio differs significantly across the genera (*p* ≤ 0.001) and pairwise comparisons reveal that *Homo* and *Pongo* are differentiated from *Pan* and *Gorilla* (*p* ≤ 0.001 for all four significant tests, Table S4).

### 3.4 Trabecular thickness

Tb.Th differs significantly across both the proximal and distal capitate of the study sample (*p* = <0.001 for both tests, Table S3). *Gorilla* has the highest mean thickness followed by *Pongo*, with *Homo* having the thinnest (Table S2). Distally, all pairwise comparisons are significant except between *Homo* and *Pan*. Proximally, *Gorilla* is differentiated from all other taxa (Figure 4c, Table S3).

*Homo*, *Pan*, and *Gorilla* have thicker trabeculae in the proximal aspect and *Pongo* in the distal aspect (Tables S4 and S5). The difference between the two segments is statistically significant for *Pan*, *Gorilla*, and *Pongo* but not for *Homo* (Figure 5c, Table S4). The Tb.Th ratio differs significantly among the study sample (*p* ≤ 0.001) and all pairwise comparisons are significant except between *Homo* and *Gorilla* (Table S4).

### 3.5 Trabecular number

Proximal and distal Tb.N differs significantly among the study sample (*p* ≤ 0.001 for both tests, Table S3). *Gorilla* has the lowest trabecular number while *Pan* has the highest number (Table S2). Distally, all pairwise comparisons are significant except between *Pongo* and *Homo*, and *Pongo* and *Gorilla*. Proximally, only *Gorilla* is differentiated from all other taxa (Figure 4d, Table S3).

The Tb.N ratio indicates that *Homo*, *Gorilla*, and *Pongo* have a higher trabecular number in the proximal aspect, and *Pan* have a higher number in the distal (Figure 5d). The differences between the proximal and distal VOI is significant for all taxa. While the Tb.N ratio differs significantly among the study sample (*p* ≤ 0.001) only *Pan* shows significant pairwise results with all other taxa (*p* ≤ 0.001 for all three significant tests, Tables S4 and S5).

### 3.6 Trabecular separation

Tb.Sp differs significantly in the distal (*p* ≤ 0.001) and proximal (*p* = 0.038, Table S3) capitate of the study sample. *Gorilla* has the most widely spaced trabeculae, while *Pan* has the most tightly packed (Table S2). Pairwise comparisons indicate that distally, *Pan* is differentiated from all other taxa (Table S3). Proximally, the only significant pairwise result is between *Pan* and *Gorilla* (Figure 4d).

The Tb.Sp ratio shows that *Homo*, *Gorilla*, and *Pongo* have greater trabecular separation in the distal capitate whereas *Pan* has greater separation in the proximal (Figure 5d, Table S5). The difference between the separation in the distal and proximal capitate is significant for all genera (Table S4). The Tb.Sp ratio differs significantly among the study sample (*p* ≤ 0.001) and the results of the pairwise comparisons mirror those of the distal segment as the only significant tests are between *Pan* and the other taxa (*p* ≤ 0.001 for the three significant tests, Table S4).

### 3.7 Total relative bone volume

**FIGURE 6:**
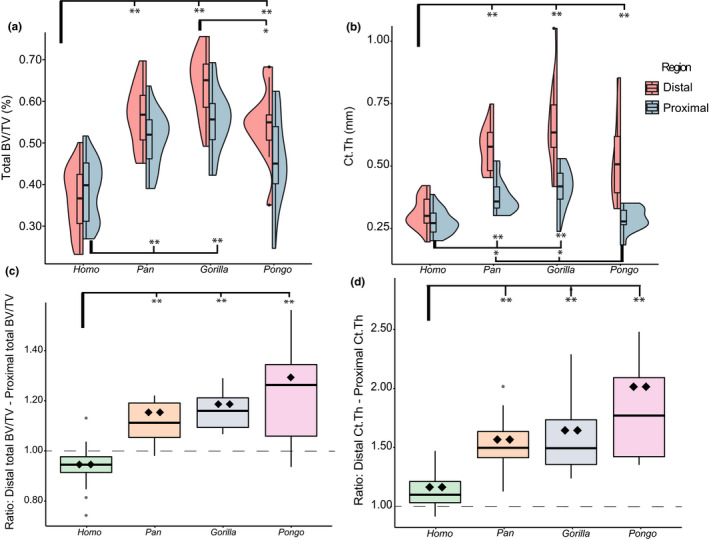
(a, b) Split violin plots showing the distribution of total BV/TV (a) and Ct.Th (b) results in the proximal and distal VOI of each genus. Images are generated using *ggplot2* in *R* (v. 1.2.1335) and utilize the default (Gaussian) kernel density estimation. Colored contours indicate the density of results across the data range. Outliers are identified with ● and represent values 1.5 times above the fourth or below the first interquartile range. Significant pairwise comparisons are indicated by the square brackets for the distal tests (top of graph) and proximal tests (bottom of graph), *p ≤ 0.05; **p ≤ 0.001. (c,d) Boxplots showing the distribution of the distal to proximal ratios of the total BV/TV (C) and Ct.Th (D) of each genus. Boxplots also show the results of the intraspecific Wilcoxon signed‐rank test and interspecific pairwise rank‐sum tests. Values above the dotted line (ratio =1) indicate greater cortical variable in the distal capitate. Significant pairwise comparisons of the ratios are indicated by the square brackets, *p ≤ 0.05; **p ≤ 0.001. Significant intraspecific Wilcoxon signed‐rank tests between the proximal and distal means are represented by the ♦ symbol thus indicate whether the difference between the mean distal and proximal trabecular variable was significantly different. ♦*p* ≤ 0.05; ♦♦*p* ≤ 0.001

Total BV/TV, which incorporates both trabecular and cortical bone, differs significantly across the study sample for both the proximal and distal capitate (*p* ≤ 0.001 for both tests, Table S3). *Gorilla* has the highest total BV/TV in both VOIs, followed by *Pan*, *Pongo*, then *Homo* (Figure 6a, Table S2). Pairwise comparisons reveal that distally, *Homo* has significantly lower total BV/TV than all other taxa (*p* ≤ 0.001 for all tests, Table S3). Proximally, the results remain the same between *Homo* and *Gorilla*, and *Homo* and *Pan*, although in this region *Homo* is undifferentiated from *Pongo*. The only significant nonhuman pairwise comparison among the proximal and distal results is in the distal VOI between *Pongo* and *Gorilla* (*p* = 0.014).

The total BV/TV ratio of the proximal and distal capitate differs significantly across the study sample (*p* ≤ 0.001). Pairwise comparisons reveal that *Homo* is differentiated from all nonhuman apes (*p* ≤ 0.001 for all significant tests, Table S4) while the nonhuman apes are not differentiated from one another (*p* = 0.51 for all three tests) (Figure 6c, Table S4). The Wilcoxon signed‐rank tests indicate that the differences in the total BV/TV between the two segments is statistically significant for all genera. As outlined in the above section, trabecular BV/TV is consistently higher in the proximal segment compared to the distal segment in all genera (Figure 4a, Table S2). However, when total BV/TV is measured, *Pan*, *Gorilla*, and *Pongo* show significantly higher values in the distal capitate (Figure 6a, Tables S2 and S4). In contrast, *Homo* maintains the trabecular BV/TV pattern, with higher total BV/TV in the proximal segment.

In the proximal capitate, the ratio of cortical bone to trabecular bone is similar among all genera, and pairwise comparisons reveal no significant results (Tables S4 and S5). In this segment, the inclusion of cortical bone increases BV/TV by 24% in *Gorilla*, 29% in *Pan*, 28% in *Homo*, and 24% in *Pongo*. Conversely, in the distal capitate the ratio of cortical bone to trabecular bone is statistically differentiated among the study sample (*p* ≤ 0.001). Pairwise comparisons indicate this is driven by *Homo*, as the cortical bone represents a significantly lower proportion of total BV/TV compared to all other nonhuman apes (Table S4). The relative portions of distal cortical and trabecular bone are similar among the nonhuman apes with cortical bone contributing 59% of total BV/TV in *Pan* and *Pongo* and 58% for *Gorilla*. In *Homo*, cortical bone represents 38% of distal total BV/TV.

### 3.8 Cortical bone thickness

Ct.Th differs significantly among the study genera in both proximal and distal capitate (*p* ≤ 0.001 for both tests, Table S3). In both segments *Gorilla* has the thickest mean cortical bone, followed by *Pan*, *Pongo*, and finally *Homo* (Figure 6b, Table S2). In the distal capitate, *Homo* has significantly thinner Ct.Th than the nonhuman apes (*p* ≤ 0.001 for all tests, Table S3), while the nonhuman apes are not differentiated from one another. In the proximal capitate, *Homo* has significantly thinner cortical bone than *Pan* and *Gorilla* (*p* ≤ 0.001) but is undifferentiated from *Pongo* (*p* = 0.386). Across the nonhuman apes, *Pongo* has significantly thinner cortical bone than *Gorilla* and *Pan* (*p* = 0.001 for both).

**FIGURE 7:**
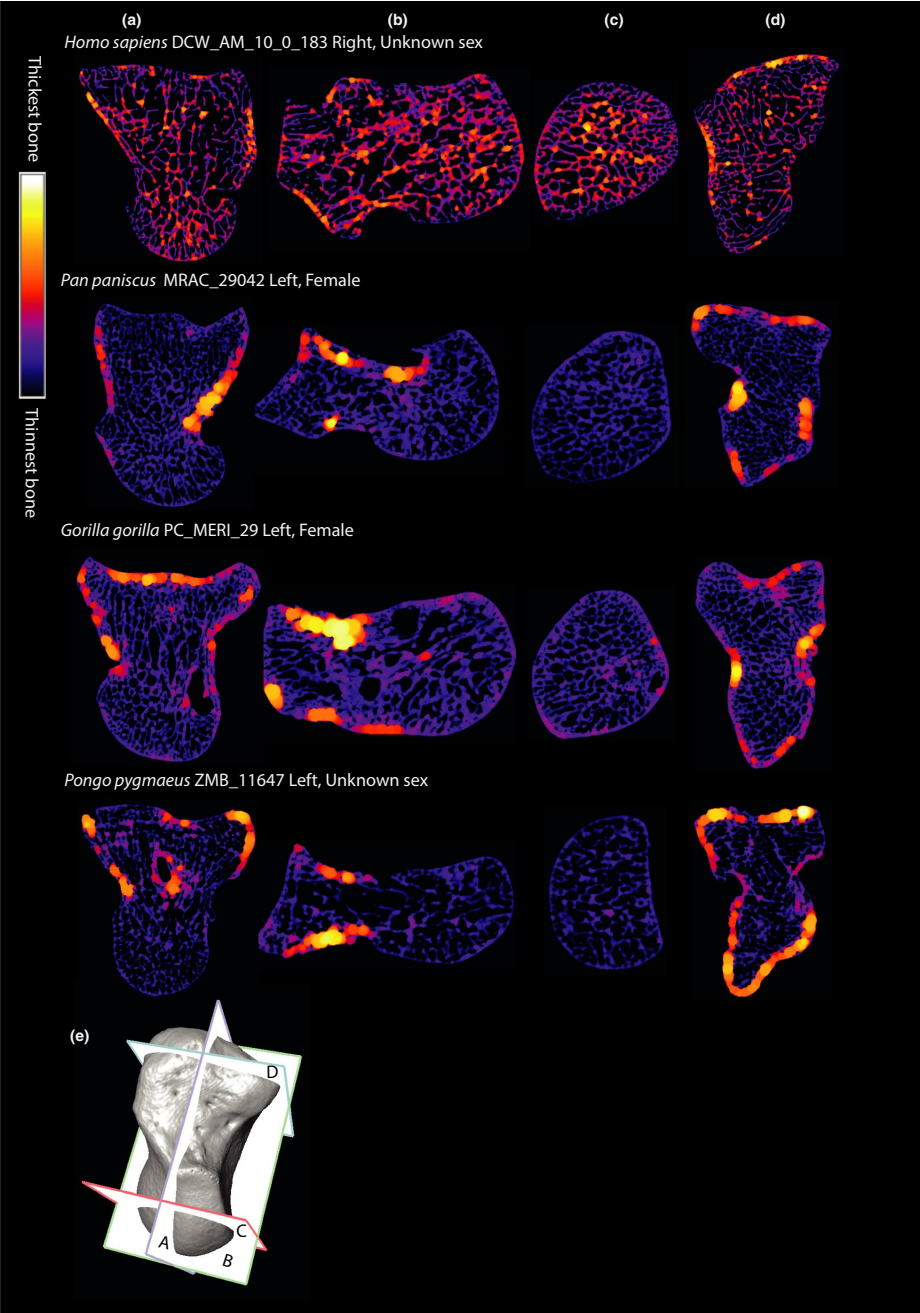
Cross‐sections from representative individuals of each genus showing relative trabeculae and cortex thickness across the capitate. (a) *Y*–*Z* dimension, radio‐ulnar cross‐section. Distal is up; dorsal is left. (b) *X*–*Z* dimension, dorsal‐palmar cross‐section. Ulnar is up; distal is left. (c) *X*–*Y* dimension, proximal‐distal cross‐section. Cross‐section taken at the proximal mid‐capitate. Dorsal is up; radial is left. (d) *X*–*Y* dimension, proximal‐distal cross‐section. Cross‐section taken at the distal capitate. Dorsal is up; ulnar is left. (e) Shows the positions of cross‐sections (a–d) on a *Pan* specimen. Left capitates have been mirrored. Capitates not to scale

All genera have thicker cortical bone in the distal VOI and the difference between the proximal and distal segments is statistically significant in all genera (*p* ≤ 0.001 for all tests) (Figure 6d, Tables S4 and S5). *Pongo* has the greatest relative cortical thickening in the distal VOI with the distal cortex being 79% thicker than the proximal, followed by *Gorilla* (62% thicker), *Pan* (52% thicker) and finally *Homo* (12% thicker). Pairwise comparisons of the ratio indicate that *Homo* is differentiated from all nonhuman apes (*p* ≤ 0.001 for all tests, Table S4). There are no significant pairwise comparisons between the nonhuman apes. The relative thickness of the cortex and trabeculae is visualized in Figure 7. In nonhuman apes, the thickest bone is consistently seen within the distal cortex. In *Homo*, the cortex and trabeculae have a similar thickness across the entire bone.

### 3.9 Allometry

The results of the allometry tests are reported in Table 3 and a figure plotting the regressions is provided in Figure S2. Trabecular and total BV/TV show a significant positive allometric relationship with capitate volume across hominoids; however, there are no significant allometric trends intraspecifically. In all interspecfic and intraspecific tests, DA is independent of capitate volume. Tb.Th shows significant positive allometry across the hominoids as well as in *Homo* and *Pongo*. In *Gorilla*, Tb.Th scales with isometry and in *Pan* it is uncorrelated. Across hominoids, Tb.N scales with negative allometry. Intraspecifically, only *Gorilla* has a significant relationship with Tb.N, scaling with negative allometry. Tb.Sp scales with positive allometry across hominoids. Intraspecifically only *Gorilla* has a significant relationship with Tb.Sp, scaling with positive allometry. Ct.Th scales with positive allometry across the hominoids, as well as in *Homo*, *Gorilla*, and *Pongo*.

**TABLE 3:** RMA regression results of the interspecific and interspecific allometry

	Variable	Isometric slope	Slope	CL−	CL+	*r* ^2^	Intercept	*p* value	Allometry
Whole sample	BV/TV	0	1.800	1.440	2.250	0.133	−2.550	**0.001**	**Positive**
	DA	0	−0.759	−0.966	−0.596	0.005	0.310	0.559	Uncorrelated
	Tb.Th	1	1.480	1.240	1.770	0.460	−2.280	**<0.001**	**Positive**
	Tb.N	0	−1.140	−1.390	−0.940	0.362	1.360	**<0.001**	**Negative**
	Tb.Sp	1	1.290	1.040	1.600	0.187	−1.680	**<0.001**	**Positive**
	Total BV/TV	0	1.830	1.460	2.280	0.150	−2.420	**0.001**	**Positive**
	Ct.Th	1	2.430	1.980	2.980	0.278	−3.160	**<0.001**	**Positive**
*Homo*	BV/TV	0	2.600	1.760	3.840	0.095	−3.510	0.125	Uncorrelated
	DA	0	1.190	0.799	1.760	0.068	−1.880	0.198	Uncorrelated
	Tb.Th	1	1.970	1.370	2.810	0.244	−2.860	**0.010**	**Positive**
	Tb.N	0	−1.630	−2.420	−1.100	0.066	1.900	0.205	Uncorrelated
	Tb.Sp	1	1.990	1.320	2.990	0.009	−2.440	0.629	Uncorrelated
	Total BV/TV	0	2.160	1.450	3.210	0.064	−2.880	0.212	Uncorrelated
	Ct.Th	1	1.980	1.370	2.870	0.194	−2.760	**0.024**	**Positive**
*Pan*	BV/TV	0	−1.770	−3.140	−0.998	0.070	1.550	0.341	Uncorrelated
	DA	0	1.390	0.785	2.470	0.070	−2.160	0.344	Uncorrelated
	Tb.Th	1	−1.520	−2.710	−0.850	0.068	1.100	0.384	Uncorrelated
	Tb.N	0	1.140	0.628	2.060	<0.001	−1.170	0.976	Uncorrelated
	Tb.Sp	1	1.480	0.823	2.680	0.011	−1.950	0.071	Uncorrelated
	Total BV/TV	0	−1.850	−3.310	−1.030	0.032	1.800	0.536	Uncorrelated
	Ct.Th	1	−2.230	−4.000	−1.250	0.040	2.230	0.493	Uncorrelated
*Gorilla*	BV/TV	0	−1.010	−1.720	−0.597	0.064	0.859	0.342	Uncorrelated
	DA	0	0.712	0.418	1.210	0.050	−1.450	0.403	Uncorrelated
	Tb.Th	1	0.959	0.678	1.360	0.618	−1.650	**<0.001**	**Isometry**
	Tb.N	0	−1.220	−1.670	−0.895	0.693	1.490	**<0.001**	**Negative**
	Tb.Sp	1	1.450	1.020	2.070	0.602	−1.940	**<0.001**	**Positive**
	Total BV/TV	0	−0.833	−1.430	−0.484	0.011	0.802	0.698	Uncorrelated
	Ct.Th	1	1.670	1.080	2.560	0.402	−2.280	**0.008**	**Positive**
*Pongo*	BV/TV	0	2.570	1.410	4.690	0.077	−3.320	0.358	Uncorrelated
	DA	0	−1.180	−2.010	−0.690	0.291	0.761	0.057	Uncorrelated
	Tb.Th	1	1.690	1.010	2.850	0.328	−**2.440**	**0.040**	**Positive**
	Tb.N	0	−1.030	−1.880	−0.567	0.079	1.180	0.351	Uncorrelated
	Tb.Sp	1	1.330	0.711	2.470	<0.001	−1.660	0.980	Uncorrelated
	Total BV/TV	0	2.040	1.160	3.580	0.203	−2.560	0.123	Uncorrelated
	Ct.Th	1	3.250	2.000	5.280	0.425	−**3.940**	**0.015**	**Positive**

Note

CL− and CL+ indicate the 95% lower and upper limits for the confidence interval. Significant test are in bold.

## 4 DISCUSSION

This study quantified the internal bone structure of the hominoid capitate using a whole‐bone methodology to examine (1) whether relative and absolute differences in trabecular and cortical parameters across hominoid taxa could be correlated to inferred habitual behavior and (2) how the parameters differed interspecifically and intraspecifically across the proximal and distal portion of the capitate.

### 4.1 Allometry in the capitate

Interspecifically, the predictions for Tb.N and DA were supported while all others were rejected. The two parameters most strongly correlated with size were Tb.Th and Tb.N. This was particularly true for *Gorilla*, which had relatively strong positive scaling for Tb.Th, Tb.N and Tb.Sp, with r‐squared values between 0.60 and 0.69. This suggests these parameters may be linked to sexual dimorphism, which is extreme in *Gorilla* (Smith & Jungers, 1997). Indeed, the largest Tb.Th and Tb.Sp, and smallest Tb.N values among the Gorillas were from males. *Pan* was the only genus that did not report at least one significant intraspecific allometric test. This indicates that capitate size differences (as a proxy for body mass differences) between *Pan troglodytes* and *Pan paniscus* have not influenced results.

The positive relationship found in BV/TV does not corroborate results of either previous study on allometry in the primate capitate (Ragni, 2020; Schilling et al., 2014) or the talus (Tsegai et al., 2017). Differences in results between this study and others may be driven by the variation in the methodologies for calculating size or body mass. While this study used the cube root of the capitate, other studies have used the geometric mean (Schilling et al., 2014; Tsegai et al., 2017), body mass (Barak et al., 2013; Cotter et al., 2009), or linear dimensions of the bone (Ryan & Shaw, 2013). Furthermore, this study used a whole‐bone mean of trabecular parameters whereas other studies have used a VOI sampling sphere (Cotter et al., 2009; Ragni, 2020; Ryan & Shaw, 2013; Schilling et al., 2014). Results are likely also affected by the species constituting the study sample or the bone used for analysis (Doube et al., 2009; Ruff, 1987; Ryan & Shaw, 2013; Tsegai et al., 2017). Nevertheless, as BV/TV is widely reported as being independent of body mass/size, results here may indicate carpals are more likely than other skeletal elements to increase BV/TV in response to size, across hominoids. However, given the similarity in capitate size between *Homo*, *Pan* and *Pongo*, the positive relationship found here is likely driven by the larger size of *Gorilla*, rather than reflecting a hominoid trend.

Ct.Th also scaled positively with size across hominoids and within *Homo*, *Gorilla*, and *Pongo*. Notably, the *r* ^2^ value for *Pongo* and *Gorilla* was high relative to other significant tests with 0.42 and 0.40 reported, respectively. These results may reflect sexually dimorphism in *Gorilla*, as the highest Ct.Th values were all found in males; however, the results were not so clear‐cut in *Pongo*, with females represented within some of the highest values. The four highest Ct.Th values in *Homo* were male; however, there was a large number of specimens with unknown sex. These results, particularly the relative strength of the *r*‐squared value, deviate from other Ct.Th studies that, for example, reported isometry in the lumbar vertebrae (Fajardo et al., 2013), positive allometry with confidence intervals incorporating isometry in the femoral neck (Demes et al., 2000) or negative allometry in the radius and humerus (Doube et al., 2009).

BV/TV and Ct.Th are a primary component of bone strength and are thus critical to inferring function and functional adaptation from form (Maquer et al., 2015). The positive allometric relationship of BV/TV and Ct.Th to size found in this study potentially limits the interpretive value of these measures. However, in both measurements the coefficient of determination was small at 0.13 and 0.27, respectively. Although the average *Gorilla* capitate volume is only 3000 cubic millimeters larger than the pooled average of the other taxa, the significant results may be strongly driven by this size difference. While the significant allometric relationships of Tb.Th, Tb.N and Tb.Sp are notable, these measures are highly correlated with BV/TV and thus each is less important as a single measure than that of BV/TV for understanding bone strength and drawing behavioral inferences. Allometry is undoubtedly complex and not yet fully understood by bone biologists. The generally low *r* ^2^ values found here indicate that size did not exert a strong influence on bone parameters in our sample, but these somewhat unexpected results indicate allometry cannot be overlooked in multispecies comparisons.

### 4.2 Can internal bone architecture differentiate locomotor modes of hominoids?

Predictions for BV/TV were broadly supported. In trabecular and total BV/TV, knuckle‐walking African apes had the highest values, *Homo* had the lowest and *Pongo* generally fell out as intermediate between the two. These intermediate values in *Pongo* were not consistently differentiated from the other taxa. For example, although *Pongo* trabecular and total BV/TV in the distal capitate was significantly greater than that of *Homo*, it was not statistically different in the proximal capitate. This pattern was not predicted given the presumed higher forces acting on the *Pongo* capitate during locomotion compared with that of *Homo* manipulation. However, previous research has found similar results with BV/TV in *Pongo* being statistically undifferentiated from *Homo* within the capitate (Schilling et al., 2014) and other skeletal elements, including the talus (Desilva & Devlin, 2012; Tsegai et al., 2013), humerus (Kivell et al., 2018) and femur (Georgiou et al., 2019).

DA in the capitate was predicted to be highest in *Gorilla* and *Pan*, intermediate in *Homo* and lowest in *Pongo*, and results did not support this prediction. DA in the distal capitate was not significantly different between the genera, suggesting that the numerous, relatively immobile articulations within this region result in a similar DA value, irrespective of hand use. *Homo* and *Pongo* had higher DA in the proximal capitate compared to the distal segment, which statistically separated them from the knuckle‐walking taxa. High DA is correlated with strength along predictable loading trajectories within joints (Cotter et al., 2009; Hammond et al., 2018; Hart et al., 2017.) In *Homo*, DA in the proximal capitate may be explained by load predictability as the DTM constitutes the path of motion in a large proportion of daily activities (Brigstocke et al., 2014; Crisco et al., 2005; Kaufman‐Cohen et al., 2019; Moritomo et al., 2014; Schuind et al., 1994). However, the relatively high DA in the *Pongo* proximal capitate was unexpected as it was assumed that the highly mobile joint and presumed variability in wrist postures adopted during arboreal locomotion would result in diverse loading of the proximal capitate and low DA. High DA is potentially linked to methodological limitations in quantifying directionality due to high Tb.Th or low Tb.N encapsulated by the sampling sphere (Dunmore et al., 2019). However, in this study, *Pongo* Tb.N and Tb.Th were intermediate between *Gorilla* and *Pan*, and thus, this result is unlikely a consequence of methodological limitations. Although some trabecular functional adaptation studies have found low DA values for *Pongo* as predicted (Georgiou et al., 2018; Kivell et al., 2018; Matarazzo, 2015; Tsegai et al., 2013) others have also found higher than expected values (Dunmore et al., 2019; Georgiou et al., 2019). Although arboreal locomotion is associated with mobile joints capable of receiving load from multiple directions, our knowledge of *Pongo* hand and wrist kinematics and kinetics is limited (but see Orr, 2010, 2017, 2018). The few studies of captive apes have provided invaluable data on the kinematics of vertical climbing (Isler, 2005; Isler & Thorpe, 2004) and quadrupedal walking (Finestone et al., 2018; Watson et al., 2011), but these behaviors constitute a small proportion of the *Pongo* locomotor repertoire (Cant, 1987; Thorpe & Crompton, 2006). Additionally, we currently lack manual pressure research on *Pongo* similar to that by Wunderlich and Jungers (2009) or Matarazzo (2013) on African apes. This research landscape may be limiting our ability to predict and interpret functional adaptation in the wrist and hand of wild *Pongo*. Nevertheless, the DA results here indicate that *Pongo* may have less variation in its wrist or hand postures than predicted with bone aligning to high loads from a low number of habitual postures.

The significantly more isotropic structure in the proximal capitate of knuckle‐walkers was also unexpected as the low range of extension during knuckle‐walking was assumed to result in high DA. Nevertheless, the DA results are contained within the range of values reported by Ragni (2020) for the *Gorilla* and *Pan* proximal capitate. Dunmore et al. (2019) similarly found the subarticular trabecular structure of the metacarpophalangeal joint in African apes to be more isotropic than predicted. While African apes are categorized as terrestrial knuckle‐walkers, they also utilize arboreal substrates variably across their lifetimes to nest and exploit high quality food resources (Neufuss et al., 2017; Remis, 1995; Thorpe & Crompton, 2006). The isotropic structure may be a reflection of diverse hand postures and loading patterns from their mixed terrestrial and arboreal locomotor repertoire. It is possible these isotropic results are an artefact of high BV/TV lowering overall DA measurements and indeed in this study the lower proximal BV/TV values of *Homo* and *Pongo* are associated with higher DA. However, the similar DA values in the distal capitate, despite diverse BV/TV values, suggests our method is able to capture variation in DA across a range of BV/TV values.

This study also investigated potential differences in ratios of bone parameters across the proximal and distal capitate, testing the null hypothesis that these ratios would be similar across hominoids. This hypothesis was generally not supported as only two ratios were statistically similar across all genera: distal trabecular BV/TV relative to proximal trabecular BV/TV and proximal total BV/TV relative to proximal trabecular BV/TV. Thus, although proximal Ct.Th in *Homo* and *Pongo* was significantly thinner than that of *Pan* and *Gorilla*, the relative proportion of cortex to trabeculae is similar across all taxa. Similarly, although eight of the 12 pairwise comparisons indicated statistically different trabecular BV/TV across the taxa (Figure 4a), the way trabecular volume differs between the two segments is consistent across hominoids. Although it was not predicted that ratio calculations would differentiate locomotor groups, three ratios distinguished *Homo* from the suspensory and knuckle‐walking taxa: (1) distal total BV/TV relative to proximal total BV/TV, (2) distal total BV/TV relative to distal trabecular BV/TV, and (3) distal Ct.Th relative to proximal Ct.Th. Together, these ratios indicate that relatively low Ct.Th in the *Homo* distal capitate is distinctive compared with the thicker cortex in nonhuman apes. As Ct.Th is correlated to bone strength (Augat & Schorlemmer, 2006), the distal capitate in nonhuman apes is likely to be better able to resist fracture or failure and withstand high mechanical loads imposed upon the region.

This distinctive cortical morphology in nonhuman apes may reflect arboreal behaviors. All nonhuman apes engage in suspensory locomotion and climb vertical supports (Neufuss et al., 2017; Remis, 1995; Thorpe & Crompton, 2006), and in both behaviors the forelimbs are loaded in tension (Hanna et al., 2017; Hunt et al. 1996; Swartz et al., 1989). The distal capitate has numerous ligament attachments that induce tensional strain onto the capitate (Kijima & Viegas, 2009; Regal et al., 2020). Bones loaded in tension have a lower failure point than those loaded in compression (Caler & Carter, 1989; Pattin et al., 1996) and therefore greater BV/TV or Ct.Th would be required to prevent failure at ligament attachment sites (Doube et al., 2009).

When comparing differences in Tb.Th, Tb.N, and Tb.Sp across our study sample, results were similar to those of previous studies of different skeletal elements; *Pan* had high Tb.N and low Tb.Th and Tb.Sp, *Gorilla* showed the inverse, while *Homo* and *Pongo* were intermediate for all of these measures (Georgiou et al., 2019; Georgiou et al., 2018; Kivell et al., 2018; Komza & Skinner, 2019; Ragni, 2020; Ryan & Shaw, 2015; Scherf et al., 2013; Schilling et al., 2014). The consistent pattern within these parameters may represent systemic, rather than strongly functionally adaptive features of bone. DA and BV/TV have been shown to account for up to 98% of bone's elastic modulus (Maquer et al., 2015) and as Tb.Th, Tb.N and Tb.Sp interact via various combinations to produce BV/TV, individual measures of Tb.Th, Tb.N and Tb.Sp may be less useful for differentiating locomotor or postural modes.

### 4.3 Do the proximal and distal segments reflect divergent strain patterns across the capitate?

Given differences in the articulations and mobility between the proximal and distal capitate, we hypothesized that these regions would show statistically different bone structure. This hypothesis was broadly supported but there was only partial support for the specific predictions. With only two exceptions (*Pongo* distal BV/TV relative to proximal BV/TV, and *Homo* distal Tb.Th relative to proximal Tb.Th), bone parameters differed significantly between the proximal and distal regions. This suggests that the internal bone is subjected to different forces and functional adaptation responses across the capitate. Ct.Th, DA and BV/TV were predicted to be higher in the distal relative to the proximal capitate due to the immobility in the distal carpal row and numerous ligament attachments. Ct.Th results in all genera supported this prediction while the DA prediction was only supported for *Gorilla* and *Pan*. All genera had significantly higher trabecular BV/TV in the proximal capitate; however, due to the great cortical thickening in nonhuman apes, total BV/TV was higher in the distal capitate of *Gorilla*, *Pan* and *Pongo*. In contrast, despite a 12% increase in distal Ct.Th, *Homo* maintained significantly higher total BV/TV in the proximal capitate. These differences in bone architecture were only revealed by holistically analyzing subregions of the capitate, while whole‐bone measures or the exclusion of cortical bone, likely would have obscured or failed to pick up these trends.

While we argue that the results of this study indicate that force transfer differs across the proximal and distal capitate, additional analyses comparing different portions of the capitate are warranted to further test this conclusion. While this study averaged parameters across entire segments, bone volume distribution methods such as those used in Tsegai et al. (2013) and Tsegai et al. (2017) would allow more nuanced analysis between the regions under compression versus tension. Further, these methods would allow a deeper exploration of the biomechanical consequences of waisted versus nonwaisted capitates and whether this aspect of morphology impacts the functional independence of the proximal and distal regions.

### 4.4 The relationship between trabecular and cortical bone in the capitate

This study reveals the importance of considering both cortical and trabecular bone in functional adaptation research, rather than investigating each tissue separately. As exhibited in Figures 6 and 7, and discussed above, the cortical bone of the nonhuman ape capitate varied substantially from that of humans. Thus, the null hypothesis that the ratios of cortical to trabecular bone would be similar across the hominoids was not supported. However, there was one notable exception, namely, that all the study taxa had similar cortical to trabeculae ratios in the proximal capitate.

The differences between the proximal and distal Ct.Th across the locomotor groups provide support for the hypothesis that thick distal cortex in the nonhuman apes is a result of functional adaptation. However, research indicates modern *Homo sapiens* have systemically low BV/TV and Ct.Th, which has been hypothesized to correlate with increased sedentism after the transition to an agricultural lifestyle (Chirchir et al., 2015; Ruff et al., 2006; Ryan & Shaw, 2015; Saers et al., 2016; Tsegai et al., 2018). Thus, it would be valuable to assess the distal Ct.Th of pre‐Holocene *Homo sapiens* to further interrogate whether thick distal Ct.Th can be correlated simply with higher loading more generally, or, as hypothesized here, is related to forelimb involvement in arboreal behavior among the nonhuman apes. Further, there are important limitations to our interpretation of cortical bone functional adaptation in short bones. Although cortical bone does model its structure during adulthood in response to load, the genetic blueprint and the process of modelling during ontogeny greatly determines cortical bone geometry (Lovejoy et al., 2003; Martin et al., 1998). Investigation on the changes to cortical bone geometry as a result of functional adaptation have predominantly focused on changes at the mid‐shaft of long bones (for examples and summary see Ruff et al., 2006 and references therein). In short bones there is unlikely to be the same capacity for the cortical bone to substantially change its geometry with modelling processes because, unlike the diaphysis of a long bone, there is not substantial room to expand (Martin et al., 1998). During adulthood, cortical bone commonly adapts its mechanical properties via changes to porosity, apparent mineral density or cellular anisotropy (Currey, 2002; Martin et al., 1998), changes that require different methodologies to assess (e.g., histology). Finally, when segmenting different bone tissues, it can be challenging to identify the boundary between cortex and trabeculae, particularly when the cortex is porous or trabeculae are especially thick. This was a particular challenge in some of the nonhuman ape capitate specimens (see Figure S1) and will likely be a limitation for many short bones, depending on the question being addressed.

## 5 CONCLUSION

The capitate of knuckle‐walking African apes and suspensory *Pongo* was differentiated from bipedal *Homo*, primarily, by thick distal cortical bone. African apes were further differentiated from *Pongo* and *Homo* by relatively isotropic trabeculae in the proximal capitate, which was not expected given the (presumably) more stereotypical loading of the wrist during knuckle‐walking. However this higher than expected DA of the capitate head in *Homo* may indicate preferential alignment of trabeculae along the DTM. Although the wrist is often conceptualized as broadly being under compression or tension, the differentiated bone architecture in the proximal and distal regions of the capitate suggests that the loading environment can differ significantly even within the small bones of the carpus and highly localized functional adaptation responses may be taking place. Further, differences in cortical bone were critical for differentiating *Homo* from nonhuman apes. While an unexpected positive relationship was found between bone volume and capitate size, the low coefficient of determination indicated size did not strongly influence group differences in bone microstructure. Given the complex biomechanical environment, and our limited understanding of intercarpal motion, (particularly in nonhuman apes) functional adaptation research of the carpals should take a holistic approach, including incorporated analysis of cortical bone.

## AUTHOR CONTRIBUTIONS

EEB conceived and designed the experiments, acquired data, performed the experiments, analyzed the data, prepared figures and tables, authored first draft and reviewed drafts of the paper, approved the final draft. TLK and MMS conceived and designed the experiments, contributed data, provided analysis tools, authored and provided critical review of manuscript drafts, approved the final manuscript.

## Supporting information

Fig S1

Fig S2

Table S1

Table S2‐S5

## Data Availability

The authors confirm that the data supporting the findings of this study are available within the article and its supplementary materials.
