# Supplementary material for: Cortical and trabecular bone structure of the hominoid capitate
Source: J Anat. 2021 May 4;239(2):351–73. doi: 10.1111/joa.13437 (PMC8273598; doi:10.1111/joa.13437)
Supplement: Supplementary file 4 — Table S2‐S5 [file JOA-239-351-s003.docx]

# Supplementary Material

**Cortical and trabecular bone structure of the hominoid capitate**

Emma E. Bird, Tracy L. Kivell, and Matthew M. Skinner^.^

Supplementary Table 1. Downloadable excel file recording specimen information and individual parameter values. Values are recorded for the whole bone as well as the proximal and distal segments. The specimen column records the curatorial institutions as abbreviations; DCW = The Duckworth Collection, University of Cambridge; INDEN = Georg-August-University Goettingen, Anthropology Collection; MPI_TC = Max Planck Institute for Evolutionary Anthropology Primatology, Tai Collection; MRAC = Royal Museum for Central Africa Tervuren; NGB = University of Kent; NHMW = Natural History Museum Vienna; NMNH = National Museum Natural History Smithsonian; PC = Powell-Cotton Museum; SMF = Senckenberg Natural History Museum, Frankfurt; UNI_FL = University of Florence; ZMB = the Natural History Museum, Berlin; ZSM = Bavarian State Collection Zoology.

Supplementary Table 2. Descriptive statistics for this study. Capitate size (mm^3^) is reported for the whole bone. Trabecular and cortical bone parameters are reported for the proximal, distal and whole bone separately. ± indicates one standard deviation above and below the mean.

| **Capitate size (mm^3^)** | | *Homo* | *Pan* | *Gorilla* | *Pongo* |
| --- | --- | --- | --- | --- | --- |
| Whole bone | Mean | 2495 ± 652 | 2418 ± 505 | 5376 ± 2,403 | 2171 ± 568 |
|  | Range | 1190 – 3606 | 1670 – 3634 | 2452 – 10200 | 1452 – 3182 |
| **Trabecular BV/TV (%)** | | *Homo* | *Pan* | *Gorilla* | *Pongo* |
| Proximal | Mean | 0.29±0.07 | 0.38±0.04 | 0.43±0.06 | 0.36±0.10 |
|  | Range | 0.20-0.42 | 0.30-0.46 | 0.30-0.54 | 0.17-0.52 |
| Distal | Mean | 0.26±0.06 | 0.34±0.04 | 0.39±0.05 | 0.33±0.06 |
|  | Range | 0.14-0.35 | 0.29-0.43 | 0.28-0.48 | 0.21-0.43 |
| Whole | Mean | 0.27±0.06 | 0.36±0.04 | 0.41±0.05 | 0.34±0.07 |
|  | Range | 0.16-0.35 | 0.30-0.44 | 0.29-0.50 | 0.20-0.47 |
| **DA (0-1)** | | *Homo* | *Pan* | *Gorilla* | *Pongo* |
| Proximal | Mean | 0.30±0.04 | 0.24±0.03 | 0.24±0.03 | 0.30±0.04 |
|  | Range | 0.21-0.39 | 0.17-0.29 | 0.19-0.31 | 0.25-0.39 |
| Distal | Mean | 0.27±0.03 | 0.26±0.02 | 0.28±0.02 | 0.27±0.02 |
|  | Range | 0.22-0.36 | 0.22-0.31 | 0.23-0.34 | 0.24-0.32 |
| Whole | Mean | 0.28±0.03 | 0.25±0.02 | 0.26±0.02 | 0.28±0.02 |
|  | Range | 0.23-0.36 | 0.20-0.29 | 0.23-0.33 | 0.24-0.32 |
| **Tb.Th (mm)** | | *Homo* | *Pan* | *Gorilla* | *Pongo* |
| Proximal | Mean | 0.23±0.05 | 0.25±0.02 | 0.34±0.04 | 0.26±0.04 |
|  | Range | 0.16-0.33 | 0.21-0.29 | 0.23-0.43 | 0.16-0.36 |
| Distal | Mean | 0.22±0.04 | 0.24±0.04 | 0.33±0.04 | 0.28±0.03 |
|  | Range | 0.16-0.33 | 0.20-0.38 | 0.24-0.39 | 0.20-0.35 |
| Whole | Mean | 0.22±0.04 | 0.24±0.02 | 0.34±0.04 | 0.27±0.03 |
|  | Range | 0.17-0.32 | 0.20-0.28 | 0.23-0.41 | 0.19-0.35 |
| **Tb.N (mm^-1^)** | | *Homo* | *Pan* | *Gorilla* | *Pongo* |
| Proximal | Mean | 1.23±0.18 | 1.25±0.10 | 1.00±0.16 | 1.19±0.10 |
|  | Range | 0.88-1.63 | 1.09-1.49 | 0.64-1.33 | 1.03-1.34 |
| Distal | Mean | 1.13±0.15 | 1.33±0.09 | 0.95±0.17 | 1.04±0.10 |
|  | Range | 0.76-1.45 | 1.19-1.56 | 0.62-1.24 | 0.82-1.17 |
| Whole | Mean | 1.16±0.16 | 1.29±0.09 | 0.97±0.16 | 1.08±0.09 |
|  | Range | 0.78-1.47 | 1.15-1.53 | 0.63-1.27 | 0.91-1.20 |
| **Tb.Sp (mm)** | | *Homo* | *Pan* | *Gorilla* | *Pongo* |
| Proximal | Mean | 0.59±0.12 | 0.54±0.05 | 0.67±0.15 | 0.57±0.07 |
|  | Range | 0.40-0.85 | 0.44-0.62 | 0.46-1.15 | 0.47-0.73 |
| Distal | Mean | 0.66±0.12 | 0.51±0.04 | 0.74±0.17 | 0.68±0.09 |
|  | Range | 0.50-1.08 | 0.43-0.58 | 0.52-1.21 | 0.58-0.91 |
| Whole | Mean | 0.64±0.12 | 0.53±0.05 | 0.71±0.16 | 0.65±0.07 |
|  | Range | 0.48-1.03 | 0.43-0.62 | 0.51-1.19 | 0.56-0.81 |
| **Total BV/TV (%)** | | *Homo* | *Pan* | *Gorilla* | *Pongo* |
| Proximal | Mean | 0.37±0.07 | 0.50±0.06 | 0.54±0.07 | 0.45±0.10 |
|  | Range | 0.26-0.50 | 0.38-0.62 | 0.41-0.67 | 0.24-0.61 |
| Distal | Mean | 0.35±0.07 | 0.55±0.07 | 0.62±0.07 | 0.53±0.08 |
|  | Range | 0.23-0.49 | 0.44-0.68 | 0.48-0.74 | 0.34-0.66 |
| Whole | Mean | 0.36±0.06 | 0.53±0.06 | 0.60±0.06 | 0.51±0.08 |
|  | Range | 0.25-0.46 | 0.43-0.65 | 0.47-0.71 | 0.32-0.66 |
| **Ct.Th (mm)** | | *Homo* | *Pan* | *Gorilla* | *Pongo* |
| Proximal | Mean | 0.27±0.05 | 0.37±0.06 | 0.41±0.08 | 0.28±0.04 |
|  | Range | 0.20-0.38 | 0.30-0.52 | 0.23-0.52 | 0.18-0.35 |
| Distal | Mean | 0.31±0.07 | 0.57±0.09 | 0.65±0.17 | 0.52±0.16 |
|  | Range | 0.19-0.42 | 0.45-0.74 | 0.41-1.05 | 0.32-0.61 |
| Whole | Mean | 0.30±0.05 | 0.52±0.07 | 0.60±0.14 | 0.47±0.13 |
|  | Range | 0.19-0.39 | 0.42-0.67 | 0.39-0.97 | 0.31-0.75 |

Supplementary Table 3. Results of the Kruskal-Wallis and post-hoc pairwise comparison tests of the mean parameters in the proximal and distal segments. In the pairwise comparisons table, values above the diagonal represent the pairwise results for the distal capitate and those below the diagonal represent the results of the proximal. Significant results are in bold.

| **Kruskal-Wallis** | | Proximal | | Distal | | |
| --- | --- | --- | --- | --- | --- | --- |
| Trabecular BV/TV | | **<0.001** | | **<0.001** | | |
| DA | | **<0.001** | | 0.593 | | |
| Tb.Th | | **<0.001** | | **<0.001** | | |
| Tb.N | | **<0.001** | | **<0.001** | | |
| Tb.Sp | | **0.038** | | **<0.001** | | |
| Total BV/TV | | **<0.001** | | **<0.001** | | |
| Ct.Th | | **<0.001** | | **<0.001** | | |
| **Pairwise Comparisons** | | | | | | |
| **Trabecular BV/TV** | | *Homo* | *Pan* | *Gorilla* | *Pongo* | |
| *Homo* | Proximal |  | **0.001** | **<0.001** | **0.011** | Distal |
| *Pan* |  | **<0.001** |  | **0.044** | 0.685 |  |
| *Gorilla* |  | **<0.001** | **0.047** |  | **0.040** |  |
| *Pongo* |  | 0.111 | 0.550 | 0.111 |  |  |
| **DA** | | *Homo* | *Pan* | *Gorilla* | *Pongo* | |
| *Homo* | Proximal |  | 1 | 1 | 1 | Distal |
| *Pan* |  | **<0.001** |  | 1 | 1 |  |
| *Gorilla* |  | **<0.001** | 0.846 |  | 1 |  |
| *Pongo* |  | 0.846 | **<0.001** | **<0.001** |  |  |
| **Tb.Th** | | *Homo* | *Pan* | *Gorilla* | *Pongo* | |
| *Homo* | Proximal |  | 0.278 | **<0.001** | **0.004** | Distal |
| *Pan* |  | 0.072 |  | **<0.001** | **0.004** |  |
| *Gorilla* |  | **<0.001** | **<0.001** |  | **0.007** |  |
| *Pongo* |  | 0.069 | 0.650 | **<0.001** |  |  |
| **Tb.N** | | *Homo* | *Pan* | *Gorilla* | *Pongo* | |
| *Homo* | Proximal |  | **0.001** | **0.004** | 0.173 | Distal |
| *Pan* |  | 1 |  | **<0.001** | **<0.001** |  |
| *Gorilla* |  | **0.001** | **<0.001** |  | 0.173 |  |
| *Pongo* |  | 1 | 1 | **0.004** |  |  |
| **Tb.Sp** | | *Homo* | *Pan* | *Gorilla* | *Pongo* | |
| *Homo* | Proximal |  | **<0.001** | 0.290 | 0.580 | Distal |
| *Pan* |  | 0.630 |  | **<0.001** | **<0.001** |  |
| *Gorilla* |  | 0.450 | **0.020** |  | 0.580 |  |
| *Pongo* |  | 0.980 | 0.980 | 0.180 |  |  |
| **Total BV/TV** | | *Homo* | *Pan* | *Gorilla* | *Pongo* | |
| *Homo* | Proximal |  | **<0.001** | **<0.001** | **<0.001** | Distal |
| *Pan* |  | **<0.001** |  | 0.034 | 0.519 |  |
| *Gorilla* |  | **<0.001** | 0.202 |  | **0.014** |  |
| *Pongo* |  | 0.112 | 0.220 | 0.089 |  |  |
| **Ct.Th** | | *Homo* | *Pan* | *Gorilla* | *Pongo* | |
| *Homo* | Proximal |  | **<0.001** | **<0.001** | **<0.001** | Distal |
| *Pan* |  | **<0.001** |  | 0.360 | 0.360 |  |
| *Gorilla* |  | **<0.001** | 0.386 |  | 0.110 |  |
| *Pongo* |  | 0.386 | **0.001** | **0.001** |  |  |

Supplementary Table 4. Results of the nine ratios and the associated inter- and intraspecific Wilcoxon tests. The results of the ratios are shown within the grey shaded cells on the diagonal. Results above 1 indicate the parameter is higher in the distal segment. Asterisks within these cells specify the results of the intraspecific Wilcoxon signed-rank test indicating whether the proximal and distal results are significantly different from one another; * = p ≤ 0.05; ** = p ≤ 0.001. Below the diagonal, the ratio values are the results of the interspecific pairwise comparisons of the ratio. Significant results are in bold. Descriptive statistics of the ratios can be found in Supplementary Table 5.

| Segment (distal/proximal) differences | | *Homo* | *Pan* | *Gorilla* | *Pongo* |
| --- | --- | --- | --- | --- | --- |
| Trabecular BV/TV | *Homo* | 0.87** |  |  |  |
|  | *Pan* | 1 | 0.90** |  |  |
|  | *Gorilla* | 1 | 1 | 0.91** |  |
|  | *Pongo* | 1 | 1 | 1 | 0.95 |
| DA |  | *Homo* | *Pan* | *Gorilla* | *Pongo* |
|  | *Homo* | 0.91** |  |  |  |
|  | *Pan* | **<0.001** | 1.09* |  |  |
|  | *Gorilla* | **<0.001** | 0.283 | 1.15** |  |
|  | *Pongo* | 0.981 | **0.001** | **<0.001** | 0.90* |
| Tb.Th |  | *Homo* | *Pan* | *Gorilla* | *Pongo* |
|  | *Homo* | 0.99 |  |  |  |
|  | *Pan* | **0.001** | 0.92* |  |  |
|  | *Gorilla* | 0.184 | **0.041** | 0.96* |  |
|  | *Pongo* | **0.041** | **<0.001** | **0.005** | 1.07* |
| Tb.N |  | *Homo* | *Pan* | *Gorilla* | *Pongo* |
|  | *Homo* | 0.92** |  |  |  |
|  | *Pan* | **<0.001** | 1.06** |  |  |
|  | *Gorilla* | 0.513 | **<0.001** | 0.94* |  |
|  | *Pongo* | 0.093 | **<0.001** | **0.039** | 0.87** |
| Tb.Sp |  | *Homo* | *Pan* | *Gorilla* | *Pongo* |
|  | *Homo* | 1.12** |  |  |  |
|  | *Pan* | **<0.001** | 0.94** |  |  |
|  | *Gorilla* | 0.788 | **<0.001** | 1.11* |  |
|  | *Pongo* | 0.356 | **<0.001** | 0.498 | 1.20** |
| Total BV/TV |  | *Homo* | *Pan* | *Gorilla* | *Pongo* |
|  | *Homo* | 0.94** |  |  |  |
|  | *Pan* | **<0.001** | 1.11** |  |  |
|  | *Gorilla* | **<0.001** | 0.51 | 1.15** |  |
|  | *Pongo* | **<0.001** | 0.51 | 0.51 | 1.21* |
| Ct.Th |  | *Homo* | *Pan* | *Gorilla* | *Pongo* |
|  | *Homo* | 1.12** |  |  |  |
|  | *Pan* | **<0.001** | 1.52** |  |  |
|  | *Gorilla* | **<0.001** | 0.810 | 1.62** |  |
|  | *Pongo* | **<0.001** | 0.380 | 0.400 | 1.79** |
| BV/TV (total/trabecular) differences | | *Homo* | *Pan* | *Gorilla* | *Pongo* |
| Proximal | *Homo* | 1.28 |  |  |  |
|  | *Pan* | 1 | 1.29 |  |  |
|  | *Gorilla* | 0.22 | 0.29 | 1.24 |  |
|  | *Pongo* | 0.36 | 0.31 | 1 | 1.24 |
|  |  | *Homo* | *Pan* | *Gorilla* | *Pongo* |
| Distal | *Homo* | 1.38 |  |  |  |
|  | *Pan* | **<0.001** | 1.59 |  |  |
|  | *Gorilla* | **<0.001** | 1 | 1.58 |  |
|  | *Pongo* | **<0.001** | 1 | 1 | 1.59 |

Supplementary Table 5. Descriptive statistics for the nine ratios calculated in this study. ± indicates one standard deviation above or below the mean.

| Regional (distal/proximal) differences | | *Homo* | *Pan* | *Gorilla* | *Pongo* |
| --- | --- | --- | --- | --- | --- |
| Trabecular BV/TV | Mean | 0.87±0.07 | 0.90±0.03 | 0.90±0.04 | 0.94±0.14 |
|  | Range | 0.64-1.00 | 0.82-0.96 | 0.82-1.20 | 0.71-1.20 |
| DA | Mean | 0.91±0.10 | 1.09±0.13 | 1.15±0.12 | 0.92±0.10 |
|  | Range | 0.69-1.14 | 0.92-1.44 | 0.95-1.41 | 0.73-1.11 |
| Tb.Th | Mean | 0.99±0.06 | 0.92±0.04 | 0.96±0.04 | 1.07±0.06 |
|  | Range | 0.83-1.19 | 0.82-0.98 | 0.90-1.04 | 0.93-1.20 |
| Tb.N | Mean | 0.92±0.05 | 1.06±0.03 | 0.94±0.07 | 0.87±0.06 |
|  | Range | 0.78-1.00 | 1.00-1.11 | 0.83-1.19 | 0.73-0.97 |
| Tb.Sp | Mean | 1.12±0.09 | 0.94±0.03 | 1.11±0.11 | 1.20±0.14 |
|  | Range | 0.99-1.34 | 0.89-1.00 | 0.78-1.29 | 1.01-1.51 |
| Total BV/TV | Mean | 0.94±0.08 | 1.11±0.07 | 1.15±0.05 | 1.21±0.17 |
|  | Range | 0.75-1.12 | 0.98-1.21 | 1.06-1.27 | 0.93-1.53 |
| Ct.Th | Mean | 1.12±0.13 | 1.52±0.21 | 1.62±0.40 | 1.79±0.36 |
|  | Range | 0.92-1.46 | 1.13-2.00 | 1.23-2.80 | 1.35-2.45 |
| BV/TV (total/trabecular) differences | | *Homo* | *Pan* | *Gorilla* | *Pongo* |
| Proximal | Mean | 1.28±0.06 | 1.29±0.07 | 1.24±0.06 | 1.24±0.06 |
|  | Range | 1.13-1.40 | 1.19-1.45 | 1.15-1.38 | 1.15-1.42 |
| Distal | Mean | 1.38±0.12 | 1.59±0.16 | 1.58±0.14 | 1.59±0.14 |
|  | Range | 1.25-1.66 | 1.43-1.73 | 1.41-1.78 | 1.42-1.84 |

Supplementary figure 1. Example of excluded *Pan troglodytes* specimen, accession ID: PC_ZVII_24, Powell-Cotton Museum. A) CT-derived surface model of *Pan* capitate showing the location of four cross-sections. Cross-sections show dense and porous bone is continuous throughout the entire capitate. B) *Pan* specimen visualized after medtool data collection. The whole bone is shown transparent in pink, allowing visualization of the constricted and discontinuous trabecular bone region within. Results of the medtool analysis of this specimen are given in the table.

Supplementary figure 2. Plots of the seven RMA regressions testing for allometry. Individual data points are colored by genus and the hominoid trend is indicated by the dotted black line and grey confidence interval. The log cube root of the volume (mm^3^) is seen across the x axis. A) Trabecular BV/TV; B) DA; C) Tb.Th.; D) Tb.N.; E) Tb.Sp.; E) Total BV/TV; G) Ct.Th.
